# Multiplexed, universal probe-based rare variant detection with USE-PCR

**DOI:** 10.1038/s41598-025-08814-5

**Published:** 2025-07-04

**Authors:** John Alvarado, Lucien Jacky, Dominic Yurk, Aaron Aguiar, Paul Belitz, Jerrod J. Schwartz

**Affiliations:** 1 ChromaCode Inc, Carlsbad, CA USA; 2 Asari AI, Pasadena, CA USA

**Keywords:** PCR-based techniques, Diagnostic markers, Biochemical assays

## Abstract

Polymerase chain reaction (PCR) is an essential tool in research and diagnostics but is limited by the number of resolvable targets, reliance on target-specific probes, and assay-specific data interpretation. To overcome these challenges, we introduce Universal Signal Encoding PCR (USE-PCR), a novel approach combining universal hydrolysis probes, amplitude modulation, multispectral encoding, and standardized analysis for robust, scalable target detection. Using 32 synthetic templates, USE-PCR demonstrates a mean target identification accuracy of 92.6% ± 10.7% at high template copy and 97.6% ± 4.4% at low template copy, with linear correlation coefficients of 0.99 across four dPCR platforms and a dynamic range spanning four orders of magnitude. Integrating USE-PCR with RNase H-based detection chemistry enables 32 single nucleotide variants to be called simultaneously with up to 86.5% accuracy in cancer cell lines. Together, these results position USE-PCR as a transformative platform for high throughput, multiplexed analyte detection with applications in research and clinical settings.

**Supplementary Information:**

The online version contains supplementary material available at 10.1038/s41598-025-08814-5.

## Introduction

Limiting dilution was first applied to enable accurate target quantitation by single molecule PCR over three decades ago^1^. Since then, digital PCR (dPCR) has undergone a transformation, marked by the introduction of new instruments and expanding applications in oncology, reproductive health, infectious disease, environmental monitoring, and forensics. To meet growing performance demands, digital PCR platforms have improved hardware ease of use, expanded the number of color channels for analyte multiplexing, and enhanced analysis software capabilities. In addition, to address a growing demand for more information from precious samples, innovations in multiplexing methods like melt analysis^2–4^, amplitude modulation^5^, multi-spectral encoding^6^, or their combination^7,8^ have been developed. However, these approaches rely on bespoke, target-specific fluorescent probes, which complicates assay design, optimization, data analysis, and cost. Each new assay requires significant time and investment to be developed and optimized for a specific platform. This is a challenge for applications like oncology and liquid biopsy, as developing a fixed multiplex panel runs the risk of quick obsolescence as novel biomarkers are discovered, new therapies are approved, and standard of care evolves. These limitations are largely absent in massively parallel sequencing approaches, where many amplicons can be measured simultaneously and can inherently encompass new biomarkers. Furthermore, standardized reagents, processes, and informatics have made sequencing workflows highly amenable to automation and applications across indications.

To address PCR assay development and workflow challenges, there has been progress in the use of universal reporter systems to simplify assay design and enable reagent portability^9–11^, including for SNP genotyping^12^, infectious disease detection^13^, and cancer variant detection^14,15^. These approaches typically incorporate primers with synthetic tails that allow for hybridization of a universal hydrolysis probe, or they leverage a target-specific mediator probe that is cleaved to activate a set of target-independent reporter systems^16^. While promising, these methods have yet to gain widespread adoption, partly because each has only been demonstrated on a single instrument type and they have shown limited multiplexing potential. The requirement for target-specific mediator probes also imposes additional sequence limitations to the assay design and may lead to reduced multiplexing efficiency.

Even with progress in instrument capability, multiplexing, and universal reporters, the digital PCR ecosystem continues to be limited due to cross-platform assay incompatibilities. Every assay is essentially platform-specific, as the primer and probe sequences, fluorophores and quenchers, and reaction chemistries are optimized for the specific instrument partitioning conditions, excitation/emission filter sets, and signal processing algorithms in the instrument software. Data analysis is manual, user dependent, and has to be reinvented for every new assay due to differences in probe hydrolysis and PCR efficiency. Together, this has created a challenging environment to scale new content due to the substantial time, investment, and platform commitment required for any new assay to be designed, developed, and validated.

**Fig. 1:**
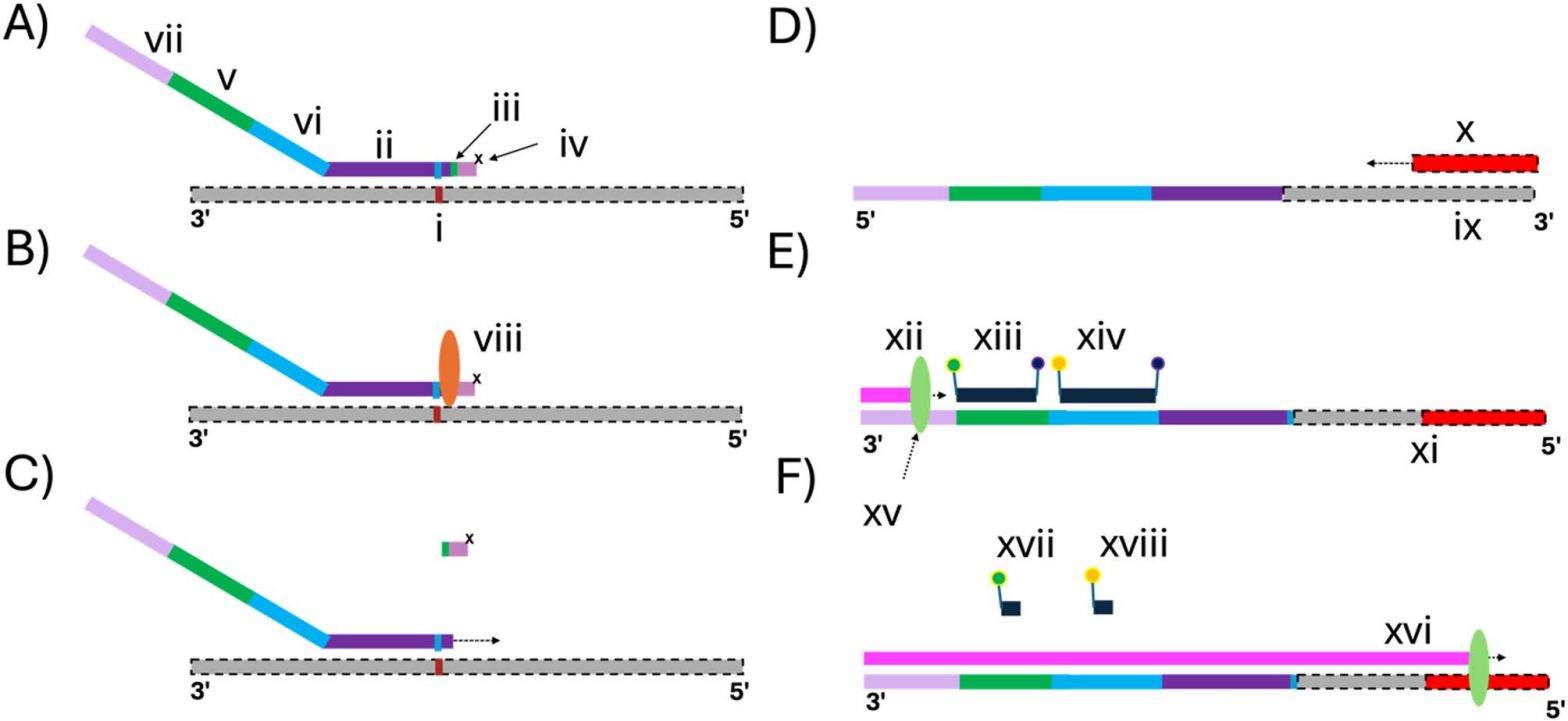
Schematic of the USE-PCR SNV detection and signal generation workflow. (**A**) An allele-specific primer (ASP) binds to a perfectly matched SNV site on a DNA template (i). The ASP includes a 3′ target-specific region (ii), a single RNA base (iii), a C3 blocker (iv), a 5′ tail encoding one or two universal probe-binding motifs (v, vi), and a universal primer-binding motif (vii). (**B**), Upon hybridization, a thermostable RNase H2 enzyme (viii) specifically digests the RNA base within the perfectly complementary DNA–RNA hybrid, removing the 3′ block. (**C**), Taq polymerase extends the unblocked ASP, generating a product (ix) that incorporates the universal motifs. (**D**) In the next cycle, a locus-specific primer (x) binds and extends the antisense strand to generate a double-stranded product (xi). (**E**) Universal primer (xii) and hydrolysis probes (xiii, xiv) bind to their corresponding motifs. In this example, Probe 1 (xiii) carries a 5′ FAM dye and 3′ quencher; Probe 2 (xiv) carries a 5′ HEX dye and 3′ quencher. (**F**) Taq polymerase (xv) extends the universal forward primer, hydrolyzing the bound probes and separating the fluorophores from their quenchers. Hydrolysis of Probe 1 (xvi) generates signal in the FAM channel, while hydrolysis of Probe 2 (xvii) produces signal in the HEX channel, enabling deterministic SNV detection encoded as 1100.

To address these challenges, we have developed USE-PCR: a novel universal probe chemistry that enables higher-order multiplexing, fast assay development, standardized data analysis, and easy portability across all leading digital PCR platforms. For single nucleotide variant (SNV) detection, USE-PCR brings together an allele-specific primer (ASP) with a 5’ synthetic tail region and a 3’ analyte-targeting region in conjunction with a locus-specific primer (LSP). The ASP’s 5’ tail is further divided into two sub-regions: a 5’ universal primer sequence adjacent to a color-coded tag that is responsible for signal generation (Fig. 1). The color-coded tag is comprised of one or more universal hydrolysis probe binding sites, and it is designed to leverage a probe mix with both amplitude modulation and multi-spectral encoding. Each color-coded tag, when successfully amplified in a partition, thus creates an allele-specific fluorescent signature. In this system, a color-coded tag utilizing one or two amplitude levels in combination with one or two color channels can encode up to 32 unique targets using ternary encoding (Table S1). Tag-containing ASPs and LSPs are combined with a universal probe mixture containing eight unique probes coupled to one of four fluorophores. This color-coded tag strategy thus decouples analyte detection from multiplexed signal generation, allowing the use of a once-optimized probe mixture with pre-defined fluorescent signatures to streamline assay development and data analysis. The color-coded tag sequences in the tails can simply be appended to any new set of primers for a new assay, and the tags will generate the same encoded signals. This approach offers a highly scalable and cost-effective solution for high multiplexing on digital PCR, eliminating the need for custom probe mixtures for each new target and assay.

We first show the performance of an eight probe universal mix to detect and resolve up to 32 synthetic color-coded tag templates, and we and illustrate how this signal encoding and decoding strategy is implemented on four different digital PCR platforms. Next, we integrate the universal probe approach with RNAse H-dependent single nucleotide variant (SNV) detection chemistry to create a 32 target oncology-focused variant detection assay compatible with two different dPCR platforms, and we benchmark its performance with three cancer cell lines. Finally, we describe how a previously optimized probe-based 9-target tick-borne pathogen qPCR assay can be quickly reformulated with USE-PCR color-coded tags so that it leverages a common universal probe mix and works seamlessly on digital PCR. Together, we illustrate the robustness and versatility of USE-PCR to create a standardized signal encoding and decoding ecosystem for digital PCR, while also enabling increased multiplexing that scales with the number of color channels available.

## Results

### USE-PCR signal encoding and decoding on four digital PCR platforms

To characterize the performance of the USE-PCR color-coded tag approach, we first designed synthetic templates containing only the signal-encoding tail portion of the ASP. These templates contained universal primer sites flanking one of 32 distinct color-coded tags (Fig. S1, Table S2). A leveled universal probe mix was designed for each of four digital PCR platforms: Thermo Fisher Absolute Q, Qiagen QIAcuity, BioRad QX600, and Roche Digital Light Cycler. The fluorophores and probe concentrations were tailored to match each instrument’s optical properties (Table S3). To maximize sensitivity, we report tags as present if one or more partitions generates signal that matches the expected optical signature (see Methods).

We first sought to examine the accuracy of the chemistry and algorithms in correctly assigning partitions to corresponding tag identities. All 32 synthetic tag templates were tested individually at high and low copy and in duplicate on the QIAcuity (Table S4). For each synthetic tag template, the intensity distribution of partitions corresponded closely with the expected intensity level (Fig. S2). Across all tag templates, the mean classification accuracy was 92.6% ± 10.7% at high copy (mean tag counts = 4880, N = 64 total reactions) and 97.6% ± 4.4% at low copy (mean tag counts = 200, N = 64 total reactions) (Table 1 and Table S5). The observed slight differences in classification accuracy between high and low copy number conditions on the QIAcuity platform are primarily attributable to signal crowding and broadened fluorescence amplitude distributions at higher concentrations. At elevated copy numbers, increased target co-occupancy within partitions and broader positive droplet clouds reduce the separation between signal populations, thereby increasing classification ambiguity. Additionally, the QIAcuity system differs from the other platforms evaluated in that it distributes its ~ 26,000 partitions across multiple sub-arrays—each with slightly different baseline signal characteristics. While we apply signal processing steps to normalize these differences (see Methods), residual baseline variation becomes more impactful at high copy numbers, where cloud overlap is more likely.

**Table 1:** USE-PCR sample call performance with synthetic tag templates. A common primer mix and universal probe mix were used to profile individual synthetic tag templates (N = 2 of each tag at high copy and N = 2 of each tag at low copy, for a total of 2048 tag calls each) and three mixtures of synthetic tag templates (N = 6 for each set at low copy, for a total of 192 tag calls for each set) on the QIAGEN QIAcuity platform. More detailed synthetic tag performance data is provided in Table S5.

	Tag call accuracy (average)	Tag call sensitivity (average)	Tag call specificity (average)
Individual tags, low copy	1998/2048 (97.6%)	64/64 (100%)	1934/1984 (97.5%)
Individual tags, high copy	1897/2048 (92.6%)	64/64 (100%)	1833/1984 (92.4%)
Set A tags, low copy	185/192 (96.4%)	55/60 (91.7%)	130/132 (98.5%)
Set B tags, low copy	189/192 (98.4%)	52/54 (96.3%)	137/138 (99.3%)
Set C tags, low copy	187/192 (97.4%)	64/66 (97.0%)	123/126 (97.6%)

**Fig. 2:**
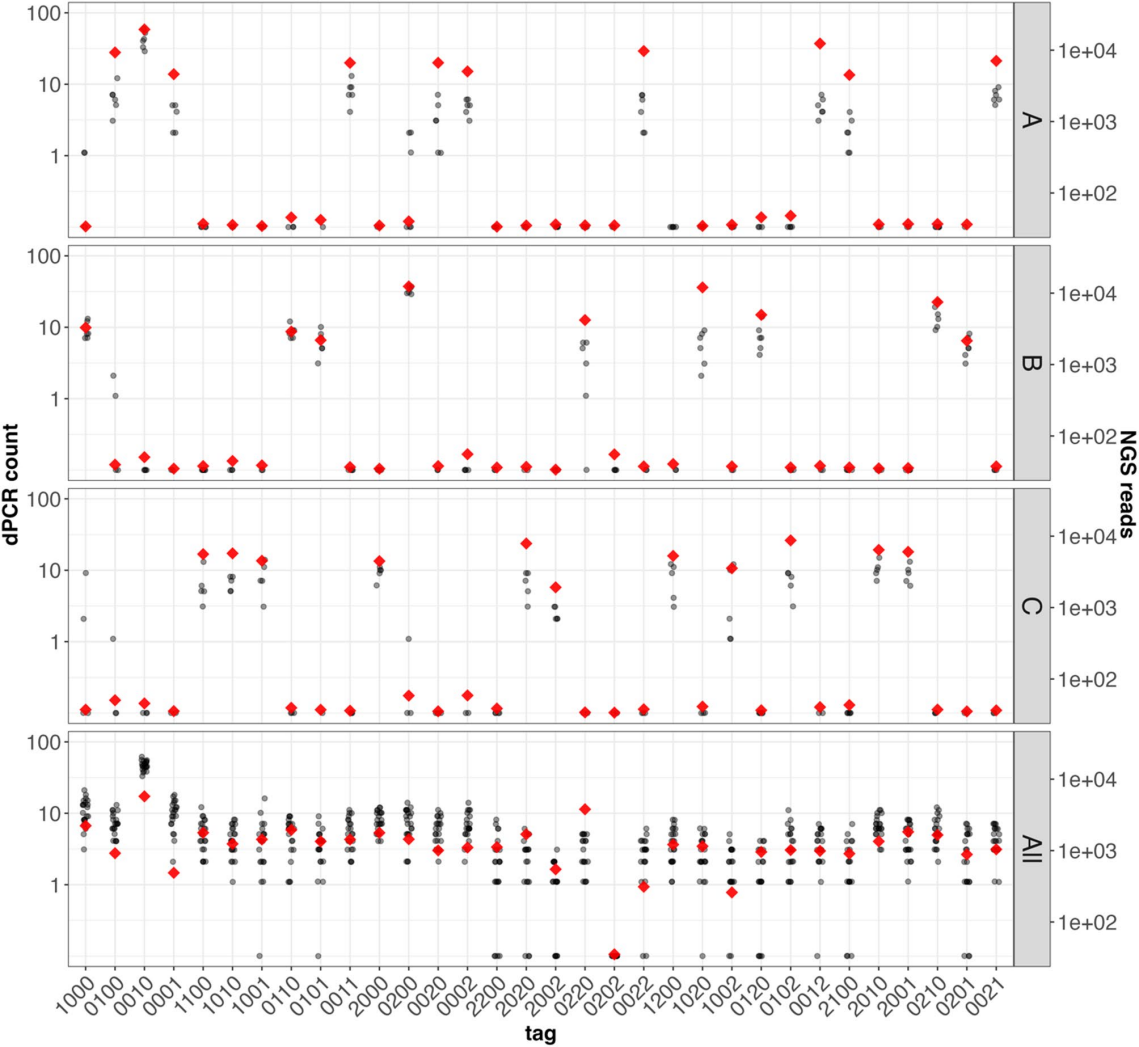
USE-PCR accurately resolves and counts targets within complex mixtures. Three mixtures each containing a subset of synthetic tag templates were profiled on the QIAGEN QIAcuity (panels A, B, C; N = 6 replicates each) along with a mixture containing 31 synthetic tag tempates (panel All; N = 20 replicates). The signal from each partition was assigned to a corresponding tag identity and total counts for each tag are given on the y-axis (grey circles, log scale). Sequencing the same samples gave the corresponding NGS reads on the right y-axis (red diamonds).

We then assembled three different groups of synthetic tag templates at low copy number (10, 9, and 11 different templates in each mix A, B, and C, respectively) and measured the tags present in separate reactions on the QIAcuity (N = 6 replicates each, Table 1 and Fig. 2). This approach allowed us to assess two key metrics: first, the fraction of false positive tags detected in a complex mixture of tags, and second, the fraction of true positive tags accurately assigned as being present in the mix. Across six replicates, USE-PCR correctly assigned 98.7%, 99.5%, and 96.8% of individual positive partitions as being present in mixes A, B, and C, respectively. The mean ± stdev tag counts in group A was 7 ± 3 counts, group B was 10 ± 3 counts, and group C was 6 ± 3 counts. False positive tag calls, which were derived from 20 partitions out of a total of 1591 positive partitions (1.3%) across all tag mixtures and replicates, were primarily attributed to incomplete probe hydrolysis, whereby a partition containing a template expected to generate an intensity “2” in a channel produced signal called as a “1”. Tag mix libraries were sequenced to confirm signal encoding tag presence, absence, and relative abundance, and the sequencing results confirmed the presence of the templates detected via dPCR.

**Fig. 3:**
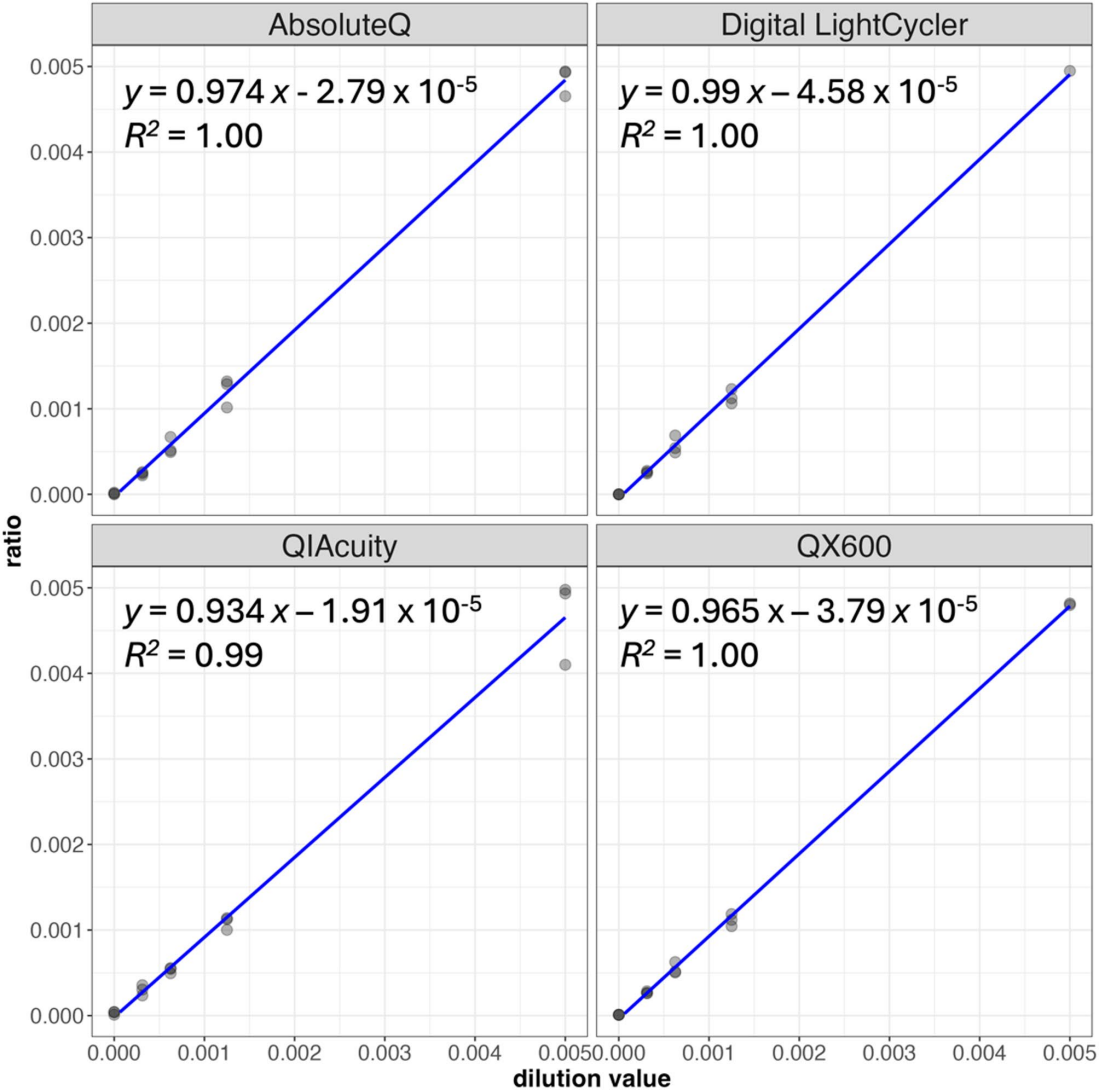
USE-PCR is portable and highly linear across different dPCR platforms. A mixture containing 32 synthetic tag templates was diluted from 0.005 to 0.0003 while one of the tags was kept at a constant copy number (N = 3 replicates at each dilution). The mixture was split and measured on four different digital PCR instrument platforms. At each dilution and on each instrument, the mean ratio of synthetic tag counts to the reference tag count is shown. Linear regression equations are shown using the least squares method.

We next examined linearity and signal encoding precision when 31 synthetic tags are present simultaneously at low copy and a reference tag is at high copy. This is a challenging situation to deconvolve, as signal is being generated in all possible amplitude and multispectral combinations. First, we diluted the 31 tag mix from 0.005 to 0.0003 with respect to the reference tag and measured the mean ratio (n = 3 replicates) on all four dPCR platforms (Fig. 3, Table S4). The mean ratio values were highly linear with regression correlation coefficients of >  = 0.99 and slopes of 0.93–0.98. At the 0.0003 ratio level, the QIAcuity gave 1.4 mean counts per tag, the Digital Light Cycler and the Absolute Q both measured 1.8 mean counts per tag, and the QX600 measured 2.3 counts per tag, which is consistent with the variation in dead volumes across instruments (Table S6). To assess the signal encoding precision when all tags are present, we examined the signal of all partitions assigned to each tag and calculated the 4D Euclidean distance between each partition’s 4D signal vector and the expected vector (Fig. S3). To minimize the impact of baseline noise in channels with no detection, signal intensities in channels without a detected tag were set to zero (for example, a partition of [0.15, 2.10, 0.20, 0.04] would be assessed as [0, 2.10, 0, 0]). Certain tags, such as 0010, had a very tight distribution and small Euclidean distances; others such as 2020 had broader and higher distance values.

We repeated the Euclidean distance analysis with synthetic tag data generated on all four dPCR platforms. Even with differences in optical hardware, partition volumes, partition mechanism, and fluorophores, some general trends emerged. First, partitions that only generated “1i” signals generally had the smallest Euclidean distance (average median distance 0.08), whereas partitions that generated signals with two “2i” signals had the highest Euclidean distance (average median distance 0.18, Fig. S4). The Roche Digital Light Cycler had the smallest average Euclidean distance across all tag classes, while the Absolute Q and the QIAcuity exhibited higher variation for tags that contained “2i” signals. This information may be helpful for assigning tags to certain targets, and to focus future efforts for tag and assay optimization. We then examined the performance of USE-PCR at detecting synthetic tags present at low copy. In the 0.0003 dilution sample, nearly all 30 of the synthetic tags were still detected in multiple replicates, and their presence was verified with parallel targeted sequencing of the tag library (Fig. S5).

To assess the long-term stability of the universal probes and their ability to generate reproducible signals, we conducted a one year storage study using a common set of universal hydrolysis probe stocks and synthetic tag templates (1000, 0100, 0010, 0001). Mean signal intensities for the ‘1i’ states remained stable across all channels between May 2024 and May 2025, with changes of − 0.8% (FAM: 9.32 → 9.25), + 10.3% (HEX: 18.9 → 20.8), − 3.0% (ROX: 21.47 → 20.83), and + 7.2% (Cy5: 17.58 → 18.84), indicating consistent detection performance over time across all channels (Fig. S6).

### 32-target USE-PCR SNV assay with synthetic templates on Thermo Absolute Q

Next, we sought to integrate USE-PCR with high multiplex SNV detection using RNAse H-dependent PCR chemistry^17^. We designed 32 USE-PCR primer systems to detect single nucleotide variants previously identified in three cancer cell lines (HCC1187, HCC1143, HCC1395; references 18, 19). For each variant, an ASP was designed with a 5’ tail region that incorporated both a universal primer site and a color-coded tag. To pair with each ASP, a LSP was designed to generate a final amplicon for each SNV of < 100 bp (Fig. 1). The allele-specific primers were blocked through incorporation of a single ribonucleotide residue followed by a 3’ blocking sequence. Activation via cleavage by RNase H2 results in increased specificity and a reduction in the formation of primer-dimers. A primer system was also designed against a background reference target (Ribonuclease P) to enable quantification of total genome copies.

**Fig. 4:**
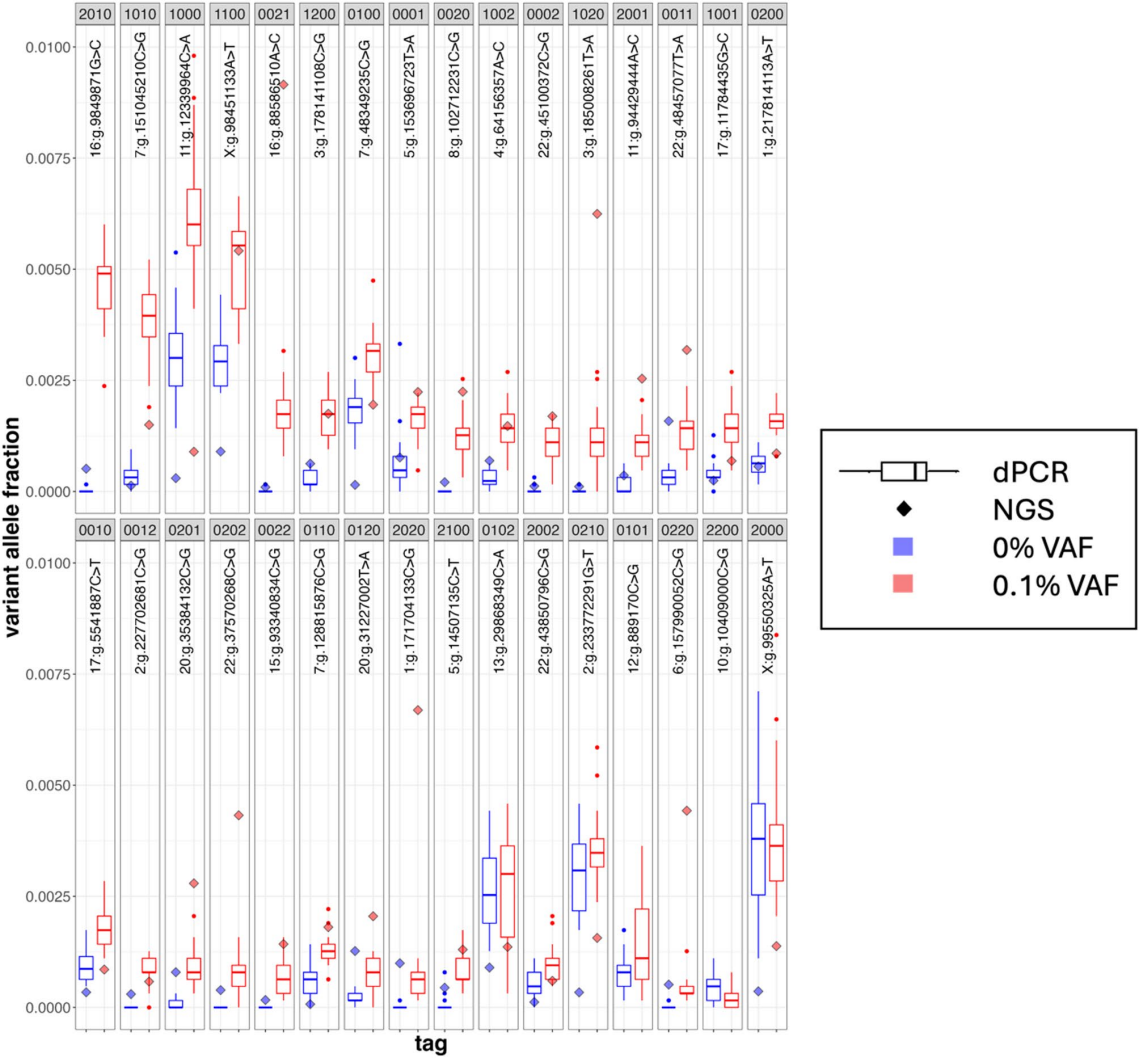
USE-PCR can detect and resolve up to 32 unique SNVs in a single reaction. A mixture of synthetic, SNV-containing templates was prepared and mixed at 0.1% dilution with a background cell line (29 ng of DNA loaded in the well, HCC1395BL). A total of 24 0% VAF replicates and 21 0.1% VAF replicates were processed on the Absolute Q. The variant allele fraction of each variant was determined with respect to a reference amplicon in the background cell line. Partitions were assigned to tags based on universal signal deconvolution (Methods section). Sequencing of both samples was performed in parallel (red diamonds), and the variant allele fraction was determined with respect to the wild type allele.

To characterize the performance of the 32 primer systems with a control sample, a set of synthetic SNV templates were designed with each containing one of the variants. During synthesis, 2 of the 32 synthetic templates failed due to sequence complexity. The remaining 30 templates were combined, diluted to 0.1% in HCC1143BL cell line genomic DNA, and 20 ng of this mixture was measured on the Absolute Q (Fig. 4). All 30 SNV targets generated positive partition counts, and 28 of the targets were statistically significantly different from the background cell line (Mann–Whitney U test/Wilcox rank-sum, *p*-value < 0.05, Table S7). Two targets (tag 2000, chrX:g.99550325A > T and tag 0102, chr13:g.29868349C > A) did not show a difference between synthetic control and wild type background, suggesting that the RNase chemistry may have certain sequence-context limitations to its specificity. The two tags for which synthetic template was not present generated low signal (tags 0101 and 2200), potentially suggesting some non-specificity for the background cell line for these SNVs. Calculating the mean VAF across all detected targets is an approach commonly used to assess tumor burden in liquid biopsy measurements; similarly, here it clearly distinguishes the 0.1% relative fraction sample and the genomic DNA background sample (Fig. S7).

### 32-plex USE-PCR SNV assay with synthetic templates on QIAcuity

To assess the portability of USE-PCR across digital PCR platforms, we then tested the same 32 USE-PCR primer mix on the QIAGEN QIAcuity with the QIAcuity universal probe mix. With the 0.1% dilution synthetic template sample, 23/30 targets were statistically significant from background, three targets were not statistically significant from background, and four targets were not detected (Fig. S8, Table S7). The QIAcuity has a higher dead volume than the Absolute Q, and so at the same molecular input and 0.1% relative fraction, we expected lower copy targets to be missed. This is further seen in the mean VAF across all SNV targets, where the delta between the contrived sample mean and background mean VAF is smaller on the QIAcuity due to there being fewer molecules to count. The 0.1% and 0% dilution samples were sequenced in parallel to confirm the presence/absence of all targets.

### 32-plex USE-PCR SNV assay with individual cancer cell line genomic DNA on QIAcuity

**Fig. 5:**
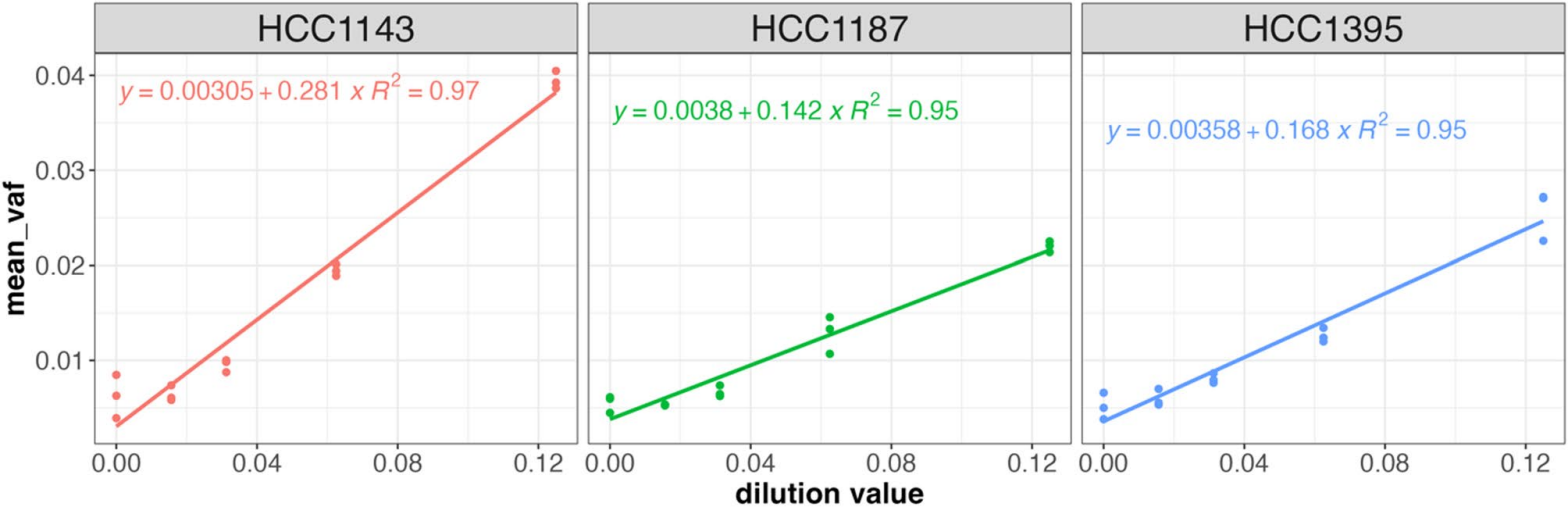
A common 32-plex primer mix and universal probe mix can be used across samples and generate linear correlations between VAF and dilution value. Three cancer cell lines (HCC1143, HCC1187, and HCC1395) were diluted in their matched normal backgrounds and profiled using a 32-plex single nucleotide variant assay on the QIAcuity (34.5 ng loaded per well, N = 3 replicates at each dilution). The variant allele frequency for each target was calculated with respect to a reference target in each replicate, and then the mean variant allele frequency across all targets is shown on the y-axis.

We then used the same 32-plex to analyze separate dilutions of HCC1143, HCC1187, and HCC1395 genomic DNA in a background of their matched normal genomic DNA on the QIAcuity. From a 12.5% to a 1.6% dilution level with a total mass input of 34 ng, all three cancer cell line samples gave linear correlation coefficients > 0.95 (N = 3 replicates, Fig. 5). Given that each cancer cell line only contained a subset of the variants in the 32-plex detection assay, we sought to assess the calling accuracy of USE-PCR on a per-partition, per-target level for each cancer line (Table S8). The overall accuracy, sensitivity, and specificity of tag assignment in this analysis reflect the combined effects of RNAse H-dependent chemistry limitations and signal encoding performance. In the 12.5% dilution samples, the proportion of positive partitions assigned to a variant known to be present (based on sequencing) was 94.6% for HCC1143, 85.1% for HCC1187, and 90.1% for HCC1395, averaged across all replicates. Accuracy for positive partitions was calculated as the number of partitions encoding targets expected to be present in the sample, divided by the total number of positive partitions.

When analyzing accuracy on a per-target basis, we measured values of 84.4%, 86.5%, and 82.3% for HCC1143, HCC1187, and HCC1395, respectively (Table 2). Per-target accuracy was defined as the number of true positive targets and true negative targets divided by the total number of targets evaluated across all reactions. We evaluated two different dPCR thresholds for calling a target as positive (> = 1 counts and >  = 5 counts) and one NGS threshold (> = 1% of reads at that position). Targets falling below these thresholds were classified as negative. In both the “by partition” and “by target” analyses, certain tags and targets were highly specific for the cell line harboring the variant of interest, with partitions only being called when that cell line was present (e.g. tag 0202, 22:g.37570268C > G was only identified in HCC1395).

**Table 2:** Target call accuracy in a 32-plex SNV USE-PCR assay. A common 32-plex primer mix and universal probe mix were used to profile six different cell lines or cell line mixtures on the QIAGEN QIAcuity platform (N = 3 replicates each; 96 total target calls for each). Mixtures were prepared by diluting cancer cell line gDNA into a matched normal gDNA background. Each of the 32 SNVs was characterized via targeted sequencing; SNVs detected at >  = 1% variant allele frequency were classified as “present” in that cell line. A sample was called “positive” for a given target if >  = 1 or >  = 5 partitions were assigned to that target.

	Partition count threshold = 1			Partition count threshold = 5		
	Target call accuracy	Target call sensitivity	Target call specificity	Target call accuracy	Target call sensitivity	Target call specificity
HCC1143 12.5%	73/96 (76.0%)	41/45 (91.1%)	32/51 (62.7%)	81/96 (84.4%)	40/45 (88.9%)	41/51 (80.4%)
HCC1187 12.5%	78/96 (81.2%)	21/24 (87.5%)	57/72 (79.2%)	83/96 (86.5%)	21/24 (87.5%)	62/72 (86.1%)
HCC1395 12.5%	79/96 (82.3%)	23/27 (85.2%)	56/69 (81.2%)	79/96 (82.3%)	21/27 (77.8%)	58/69 (84.1%)
HCC1143 0%	92/96 (95.8%)	–	92/96 (95.8%)	93/96 (96.9%)	–	93/96 (96.9%)
HCC1187 0%	92/96 (95.8%)	–	92/96 (95.8%)	93/96 (96.9%)	–	93/96 (96.9%)
HCC1395 0%	90/96 (93.8%)	–	90/96 (93.8%)	93/96 (95.8%)	–	93/96 (95.8%)

A few tags and targets showed potential error modes of USE-PCR as currently implemented. The first error mode appears where the RNase H2 chemistry lacks high SNV specificity (e.g. tag 1000 (11:g.12339964C > A), which resulted in positive partitions with all three cell lines. A second error mode appears where a tag with two probe sites generates signal slightly lower than expected for the second probe (e.g. a tag with encoding [0, 0, 1, 2] generates signal at [0, 0, 1, 1.5]), and the partition may be misassigned. A third error mode occurs when certain targets are present at high copy, and the partition cloud broadens to include rain. Tags containing a single probe site at the lower signal intensity (e.g. 1000) are the most susceptible to miscalls due to rain from tags encoded at higher intensities. The “1i” signal intensity tags are also sensitive to well-to-well or plate-to-plate variation within the negative partition cloud, which may be due to optical imaging artifacts that results in less distinct separation between the 0 and 1 intensity levels.

### Upgrading an existing probe-based multiplex qPCR assay to USE-PCR

Next, we sought to explore the potential of upgrading an existing PCR assay to leverage universal signal encoding. We had previously developed an optimized set of 9 primer systems with 9 different target-specific probes for tick borne pathogen (TBP) detection using qPCR. In this probe-based system, each target is encoded in two color channels using multispectral encoding (Fig. S9A). To re-encode the primers with USE-PCR signal encoding, color-coded tags were added to one primer, blockers were added to the 3’end of both primers, and a ‘GC’ dinucleotide was added to the 5’ end of the non-tag primer. Targets were encoded with tags to leverage the same QIAcuity universal probe mix as described above with no further optimization. Experiments with synthetic control templates illustrate the re-encoded USE-PCR primers demonstrating high specificity for their intended target (Fig. S9B), with terminal intensity values consistent with synthetic tails alone. The 5D Euclidean distance between each partition’s 5D signal vector and the expected vector showed similar results to synthetic tags, where partitions generating an intensity level of “2i” exhibited the highest Euclidean distance (average median distance: 0.33, Fig. S10). The greater variation in the “2i” signals can be attributed to the calibration process, where intensity “1i” signals were used to set the reference intensity equal to 1. Small differences in the relative concentrations of the “1i” and “2i” probes can lead to an increase in the Euclidean distance of “2i” targets. Overall, this highlights the robustness and flexibility of USE-PCR signal encoding to port existing assays from qPCR to dPCR, and points to potential benefits of standardizing a universal probe mix across different assays.

## Discussion

Here we introduce USE-PCR, which leverages a universal probe mix in conjunction with amplitude modulation and multi-spectral encoding, and show how it can enable up to 32 single nucleotide variants to be measured and accurately resolved at high sensitivity in a single digital PCR reaction. The approach offers a scalable and standardized way to create high multiplex assays on digital PCR while also reducing development time. The encoding strategy uses 8 unique probes to achieve 32 target multiplexing in 4 color channels, and a probe mix can be built once and is then compatible with any assay designed with appropriate tag-encoded tails. This enables digital PCR to have more of a library preparation-like workflow: similar to the adapter ligations that enable sequencing flowcell compatibility, the USE-PCR workflow incorporates color-coded tags to enable universal probe mix compatibility. The fluorescent dye-containing molecules, like in sequencing, are standardized and re-used from run to run and assay to assay, enabling future economies of scale in synthesis and quality control. We also show that existing PCR assays can be easily re-encoded to leverage universal signal encoding. The signaling bandwidth afforded by USE-PCR could enable assay developers to simultaneously port an existing assay while also adding in additional targets to increase the information content generated from a reaction.

Universal signal encoding is flexible to grow with new PCR instrumentation, as it can take advantage of additional color channels to further increase multiplexing. For example, tags designed with one or two probes sites on a five color channel instrument could encode up to unique targets. Similarly, a six color channel instrument leveraging tags with one or two probes could encode and resolve up to unique targets. While beyond the scope of the present work, it may be possible to incorporate more than two probe sites in the USE-PCR amplicon. For example, if up to four probe sites are incorporated in color-coding tags on a four color channel instrument with two amplitude levels, up to 3^4^–1 = 80 unique targets could be encoded in a single reaction. Similarly, with six probes on a six color channel instrument and two amplitude levels, up to 3^6^–1 = 728 unique targets are theoretically possible.

1 $$\left({}_{1 }^{5}\right)\times 2+\left({ }_{2}^{5} \right)\times {2}^{2}=50$$

2 $$\left({}_{1 }^{6}\right)\times 2+\left({ }_{2}^{6} \right)\times {2}^{2}=72$$

To examine the practical limits of USE-PCR multiplexing at higher plex, we performed Poisson simulations under different conditions relevant to digital PCR partitioning. These simulations evaluate how assay design constraints such as target concentration and panel size impact the expected frequency of signal co-presence within partitions (i.e., where two or more targets appear together). We note that these models assume ideal assay conditions—specifically, no primer–primer interactions, off-target effects, or amplification bias—as our focus is on the theoretical limits imposed by universal signal encoding and stochastic partition loading. We use a 5% co-presence threshold as a conservative benchmark, representing the point at which quantification accuracy may begin to degrade due to signal ambiguity between co-localized targets sharing the same probe. In the 32-plex assay described here, with 26,000 partitions, up to 100 copies of each target can be present before 5% of positive partitions become ambiguous. In a hypothetical 728-plex assay, this threshold is reached when approximately four copies of each target are present (Fig. S11). This level of resolution is well-suited for applications such as minimal residual disease testing, where precise tracking of tumor clone dynamics is critical for informing clinical decisions.

To evaluate the impact of uncorrected optical crosstalk on partition-level calling accuracy in highly multiplexed USE-PCR, we simulated 728-plex reactions across a range of target concentrations and crosstalk levels (0–100%). At modest crosstalk (e.g., 20%), co-presence remains the dominant factor affecting call accuracy. However, as crosstalk exceeds 50%, its influence becomes more pronounced, leading to a sharp decline in partition-level accuracy. Notably, simulations show that 0% and 20% crosstalk result in nearly identical accuracy profiles, while 80% and 100% crosstalk generate overlapping performance curves, each showing approximately a 17% reduction in accuracy at 13 copies per target (Fig. S12).

These results highlight the importance of minimizing optical crosstalk in order to preserve quantification accuracy, particularly in high-plex applications. While signal encoding strategies like USE-PCR can tolerate moderate crosstalk, achieving robust performance at scale requires careful consideration of optical and biochemical design. In parallel, alternative strategies for enhancing digital PCR multiplexing with universal reporters have also been explored. One such approach was recently described^15^, relying on target-specific, non-fluorescent probes that generate signal via a universal reporter during PCR only in the presence of the target sequence. However, the approach required extensive optimization of cycling conditions, primer/probe concentrations, and probe/reporter ratios, suggesting that scalability and portability may be limited. Separately, a method using “optical signatures” has been developed to distinguish dyes with subtly different spectral emission patterns across channels, enabling resolution of up to 22 infectious disease targets^7^. However, this approach requires labor-intensive calibration for each new assay and depends on UMAP for data dimensionality reduction, which can introduce challenges in interpretability and stability. In contrast, USE-PCR employs a fixed set of universal probes and tag sequences with deterministic, stable performance across assays, enabling logical and interpretable data analysis. The universal probe mix requires characterization only once during initial assembly, eliminating the need for per-assay calibration. Moreover, new assays can be developed without designing or validating new probes, significantly reducing development time and cost.

There are a few limitations to USE-PCR that may be addressed with future improvements to chemistry and hardware. First, the signal generation performance is partially dependent on the associated detection chemistry performance. Here, we used RNAse H-dependent PCR as an example for SNV variant detection and observed that certain tags containing two probe sites can experience slightly decreased signal from the second probe site. Primers were intentionally selected for SNV specificity and not PCR efficiency, which may contribute to this phenotype. This could be due to inefficient hydrolysis during primer extension due to amplicon secondary structure or other tag-target interactions. To address this, a different tag could be selected for that specific target, or new tag sequences could be designed to balance amplification efficiency with target specificity. USE-PCR could alternatively be integrated into alternative detection chemistries such as molecular inversion probes^20^, Invader^21^, or ARMS^22^, which could leverage different probe site configurations. A second limitation is that the excitation and emission filter sets across PCR instruments are similar but not always exactly the same. This makes the creation of a single universal hydrolysis probe mix that works optimally on every platform a potential challenge. A platform-universal probe mix might be designed to leverage only dyes shared across instruments (e.g. FAM, HEX, Cy5), and therefore tradeoff multiplex potential for instrument compatibility. A final limitation is the ability to accurately resolve many targets simultaneously when the targets are all present at high copy, due to the fixed number of partitions and Poisson statistics. This issue can potentially be addressed by splitting a high copy sample across multiple reactions. To further contextualize the strengths and trade-offs of USE-PCR relative to alternative targeted detection methods, such as massively parallel sequencing, we provide a detailed comparison in Table S9 using a hypothetical 32-target rare SNV detection scenario as an example. While massively parallel sequencing can offer deep per-SNV read coverage, it typically incurs high redundancy and requires deduplication and error correction to resolve low-frequency variants. In contrast, USE-PCR enables direct molecule counting at comparable sensitivity, while substantially reducing cost, turnaround time, and sample input requirements. USE-PCR, with a single universal probe mix, enables higher levels of multiplexing with systematic interpretation steps, and streamlines multiplex PCR assay development by eliminating variation due to target-specific probes. These are two critical features that create broader utility for digital PCR as an analytic and clinical diagnostics tool. Further advances in dPCR instrumentation, oligonucleotide synthesis, and multiplex primer design^23^ will further benefit USE-PCR strategies by making rapid, low cost, high information content assays even more accessible and useful. A key advantage of digital PCR over massively parallel sequencing is compatibility with low input or challenging samples^24^, and USE-PCR is strongly positioned to build on these advantages by making high plex assays easier and less expensive to develop. While this study focuses on technical validation using cell lines and synthetic templates, future work will extend USE-PCR to clinical samples to evaluate performance in real-world diagnostic settings.

## Materials and methods

### Universal probe design and synthesis

A total of 14 locked nucleic acid (LNA) universal probe sequences, each 13 bases long, were designed using Primer3^25^ and custom thermodynamics-based filtering. Five LNA bases were incorporated at internal probe positions only (no LNA at the 5′ or 3′ end) and limited to stretches of at most three consecutive LNA nucleotides, in line with established design guidelines to prevent excessive duplex stabilization and synthesis difficulty. The remaining probe nucleotides were standard DNA. The inclusion of five LNA monomers in a 13-mer provides a substantial increase in duplex stability, allowing short probes to attain high melting temperatures. Probes were designed to have melting temperatures (T_m_) in the narrow range of 68–73 °C to ensure uniform binding stringency across different targets.

Melting temperature predictions for candidate probe sequences were performed using the IDT OligoAnalyzer tool (version 3.1) under solution conditions mimicking PCR buffer: 0.2 µM oligonucleotide, 50 mM Na^+^, 3 mM Mg^2+^, and 0.8 mM dNTPs. A nearest-neighbor two-state model was used to calculate duplex T_m_, accounting for the thermodynamic contribution of each neighboring base pair. Nearest-neighbor parameters for standard DNA/DNA Watson–Crick pairs were taken from^26^, while LNA-containing nearest-neighbor parameters were applied for sequences with LNA modifications^27,28^. An in silico analysis was performed by generating all possible combinations of 8 probes and generating all sequence permutations of 1 and 2 probes with flanking common primers. The delta Gibbs free energy was calculated for the binding of each probe to each of the 32 tags for each set. A final set of 8 probes was selected in which the delta Gibbs free energy was > −8000 kJ/mol for all combinations of probe and tag except in cases when the probe was expected to bind to its specified tag. Probes corresponding to the selected sites were synthesized by Integrated DNA Technologies with HPLC purification, shipped lyophilized, and resuspended between 100 and 200 µM in 1X TE.

### Synthetic tag template design and synthesis

Synthetic tag template sequences were designed by concatenating a universal forward primer sequence (based on the human ACTB sequence), a first “AT” padding sequence, a first universal probe binding site, a second “AT” padding sequence, optionally a second universal probe binding site, and a universal reverse primer sequence (also based on ACTB, Table S2). The universal primer sequences were selected based on extensive prior performance characterization on digital PCR^8^. A combinatorial four color tag set, with one or two probe sequences, and one or two amplitude levels, results in 32 unique synthetic tag templates. The 32 designed tag sequences were manufactured by IDT using the pIDTSMART-AMP vector. Plasmids were shipped lyophilized at ambient temperature and resuspended overnight at 4 °C. Plasmids were then diluted to a calculated 4 × 10^2^ copies per μL and quantified in triplicate using digital PCR by measuring a common sequence in the plasmid backbone. All synthetic tag plasmid templates were combined into a single tube with 31 tags at 200 copies per μL and one tag as a background control at 2000 copies per μL. One ten-fold dilution was performed down to 1% and then the sample was serially diluted in twofold dilutions down to 0.03%.

### Individual universal probe testing with synthetic tag templates

Each universal probe was first tested individually using its corresponding synthetic tag template, which was designed to generate signal only for the specific probe under evaluation. Reactions were performed in triplicate on each instrument using the probe concentrations listed in Table S3, along with common ACTB flanking primers at 300 nM. Platform-specific mastermixes were used for each system, and all runs were carried out using the respective instrument’s standard cartridge and workflow, following the manufacturer’s recommendations. Thermal cycling conditions included the enzyme-specific activation time and temperature recommended for each platform, followed by 40 cycles of 95 °C for 20 s (denaturation) and 58 °C for 120 s (combined anneal/extension).

### Universal probe mix formulation for amplitude modulation and multispectral encoding

A 20X universal probe mix was prepared for each of the four digital PCR instruments to match the available emission filters and the dynamic range of the detectors (Table S3). For each instrument, all 8 probes were combined into a single probe mix in 1X TE. Only the 4 channel mixes were used in this step, with probes selected to match the filters listed for each instrument. Using the tag-specific templates described above, each probe was amplified individually to ascertain the amount of signal generated in each channel and intensity level. The concentration of probe at intensity level 1 was then adjusted based on the signal generated by the probe at intensity level 2 in each channel, such that the signal from Probe 1 was approximately 50% of the signal from Probe 2. This 2:1 intensity ratio was selected to enable additive signal interpretation of co-positive partitions while minimizing the risk of signal saturation.

Final probe concentration adjustments were performed for each channel on each instrument to account for differences in signal detection sensitivity, proprietary fluorescence units, and channel-specific crosstalk correction. Probe concentrations were tuned to target the lower end of each instrument’s dynamic range, where signal is most linear and comparable across platforms. This approach ensured distinguishable single-positive and multi-positive partition clusters while minimizing inter-instrument variability.

Additional considerations were made to account for differences in thermodynamic environments and partition volumes across platforms. For example, droplet-based systems like the Bio-Rad QX600 have a pre-cycling input volume of ~ 20 µL but expand to > 40 µL post-droplet generation, requiring longer thermal transitions (Table S6). In contrast, nanowell array systems such as the QIAGEN QIAcuity and Roche Digital LightCycler operate in fixed nanoliter-scale volumes with more efficient thermal cycling. To accommodate these differences, probe concentrations were optimized to promote near-complete consumption while still maintaining sufficient fluorescence signal to resolve positive partitions under the least efficient thermal cycling conditions.

### Instrument setup for universal probe mix synthetic tag measurements

The Thermo Fisher Absolute Q, QIAGEN QIAcuity, Roche Digital Light Cycler, and Bio-Rad QX600 were configured with default data collection settings according to the manufacturer. Only the expected emission filters were set to active. Plates were prepared for each instrument using the corresponding recommended PCR master mix, plate type, and preferred loading method. To enable platform to platform comparisons, 5 μL of tag mix sample was loaded into each well regardless of total reaction volume and platform dead volume differences (Table S4). A 20X primer mix was prepared by combining the forward and reverse flanking primers at 6 μM (forward primer: CCT TGC ACA TGC CGG AGA T, reverse primer: ACA GAG CCT CGC CTT TGA T). The same 20X primer mix was used across all four instruments. Each tag mix sample was loaded in triplicate. For each of these instruments the reactions were vortexed for 30 s twice and spun down for 30 s prior to being transferred into their respective cartridges or partition generators. The Absolute Q and QX600 had a 95 °C enzyme activation temperature for 10 min; the Lightcycler and QIAcuity had a 95 °C activation temperature for 2 min. This was followed by 40 cycles of 95 °C for 20 s denature step and 58 °C for 120 s combined anneal/extension step. The QX600 plate included the recommended deactivation step of 98 °C for 10 min at the end of cycling.

Raw data files from each instrument were crosstalk corrected, and data from each well was standardized by subtracting the 3rd percentile of amplitudes in each channel from all partitions. This corrected for baseline drift and ensured that all wells had a no-target partition cluster at roughly 0 in all channels. The dyes used to encode individual tags emit across broad spectral ranges, resulting in fluorescence crosstalk into neighboring detection channels. To accurately decode multiplexed signals, spectral crosstalk was removed using a compensation approach. A crosstalk matrix was first generated from single-color control reactions to quantify the extent of signal crosstalk between channels. For each partition, baseline-subtracted fluorescence intensities were arranged into a vector containing the amplitude values for each channel, which was then multiplied by the inverse of the crosstalk matrix to produce spectrally corrected data. This transformation ensured that each corrected channel value reflected only its intended fluorophore, enabling accurate and robust tag classification. Extraction of target copy numbers from these data was performed using the method described below.

### Sequencing comparison for synthetic tag templates

Synthetic tags mixtures were amplified in bulk by mixing 10 μL template (each tag at 200 copies per μL), 5 μL of the QIAcuity Master Mix, 1 μL 20X primer mix, and 4 μL water. Bulk PCR was performed on a BioRad C1000 thermal cycler with the following cycling conditions: 95 °C for 10 min, followed by 35 cycles of [95 °C for 15 s, 60 °C for 30 s, 72 °C for 60 s]. PCR products were isolated from unreacted primers using 1.8X AMPure (Beckman), and the products were then prepared for sequencing using the NEBNext Ultra II DNA Library Preparation Kit (New England BioLabs). Libraries were sequenced on an Illumina MiniSeq (2 × 150 bp reads). Raw reads were first trimmed (fastp) and then aligned to a reference file containing the tag sequences using bwa^29^. Only reads that had a perfect match to reference tag sequence were counted.

### Long term stability testing of universal probe mix with synthetic tag templates

A universal probe mix was initially created in May 2024 using stock lots of hydrolysis probes The probe mix was immediately tested with a mixture of synthetic tag templates (tags 1000, 0100, 0010, 0001) on the QIAGEN QIAcuity as described above. All stock lots of hydrolysis probes were stored at 4 °C. In May 2025 a new universal probe mix was made from the same stock lots of hydrolysis probes and tested using the same synthetic tag templates to assess for degradation in signal intensity (Fig. S11).

### Integration with rhAmp PCR for Synthetic SNV detection

Three previously sequenced cancer cell lines (HCC1143, HCC1187 and HCC1395, human genomic DNA) with both cancer and matched genomic DNA sequencing data available were obtained from ATCC^16,17^. A total of 50 single nucleotide variants for each cell line were selected for the assay. Detection primers for each variant (one allele-specific primer (ASP) and one locus-specific primer (LSP)) were designed using IDT’s rhAmp Genotyping Design Tool and ordered with a common tail. Each primer system was screened in isolation for both sensitivity and specificity on the ThermoFisher Absolute Q. The 32 best performing ASPs were then re-ordered to incorporate one of the 32 unique tags in the 5’ tail (primer sequences are provided in Table S10). A 10X ASP/LSP primer mix was made by combining all 32 ASPs, each with a unique tag, at 0.5 μM each, the 32 LSPs at 3 μM each, and the universal forward primer at 6 μM. A 40X reference dye mix was prepared with a final concentration of 1 μM unquenched ROX probe (poly(T)10) and 1% Tween-20 (Promega REF H5152).

To create a set of SNV control templates, a unique plasmid was ordered for each of the selected 32 SNVs. Plasmids were manufactured and diluted down to 2 × 10^3^ copies/μL and quantified as described above. Two of the plasmids were not able to be synthesized due the complexity of the region. The remaining 30 synthesized plasmids were combined into a single sample at 200 copies/μL. To create a background for the SNV templates, cell line stock HCC1143BL was quantified on a Qubit™ using the 1X dsDNA, high sensitivity kit (cat# Q33231). The genomic DNA was diluted to 6.9 ng/μL to create a 0% sample and background for positive samples. The combined plasmid mix was diluted in the genomic DNA in 1:10 dilutions to create the 1% and 0.1% samples.

To create the cell line DNA dilutions, all three cell lines’ matched normal and tumor DNA were quantified on a Qubit™ using the 1X dsDNA, high sensitivity kit (cat# Q33231). For individual cell line titrations, samples were diluted to 6.9 ng/μL. Cancer cell line DNA was spiked in matched normal DNA at 12.5%. Two-fold serial dilutions were performed down to 1.6% for 4 total dilutions for each cell line. The matched normal at 6.9 ng/μL was used for 0%.

For the combined cell line DNA titration, all three normal cell lines’s DNA were combined in a single tube and all cancer cell lines’ DNA were combined in a single tube at a concentration of 2.3 ng/μL per cell line. The combined cancer cell line DNA pool was spiked into the normal pool at 12.5%. Two-fold serial dilutions were performed down to 1.6% for 4 total dilutions of cell line pool. The normal pool was used for 0%.

### Cell line SNV dilution testing on Absolute Q

For each sample to be tested, a PCR mastermix was made by combining 6 μL of rhAmp Genotyping Mastermix (cat. 1,076,447), 0.3 μL of 40X universal probe mix, 1.2 μL of 10X primer mix, and 0.3 μL of 40X reference dye mix. This mixture was vortexed vigorously for 10 s and spun down to collect contents. Next, 7.8 μL of the PCR mastermix was added to each well of a 0.2 mL strip tube. 4.2 μL of sample was added to each well. Strip tubes were vortexed for 10 s and spun down twice. 9 μL of each sample was transferred from the strip tubes to the QuantStudio™ Absolute Q™ MAP16 Plate (A52865). 12 μL of QuantStudio™ Absolute Q™ Isolation Buffer was added to each well. MAP 16 plate was sealed using the provided caps and loaded onto the Absolute Q ™ Instrument.

### Cell line SNV dilution testing on QIAcuity

For each sample to be tested, a PCR mastermix was made by combining 8 μL of rhAmp Genotyping Mastermix (cat. 1,076,447), 0.4 μL of 40X universal probe mix, 1.6 μL of 10X primer mix, 0.36 μL of 45X reference dye mix, 0.36 μL of a 45X mix containing primers and probe for RNASEP and 0.29 μL of 1X low EDTA TE. This mixture was vortexed vigorously for 10 s and spun down to collect contents. Next, 11 μL of the PCR mastermix was added to each well of an Applied Biosystems 96 well plate (ref 4,483,485). 5 μL of sample was added to each well. The plate was vortexed for 10 s and spun down twice. 12 μL of each sample was transferred from the 96 well plate to a 96 well QIAcuity Nanoplate 8.5 k (cat 250,021). The plate was sealed with the included nanoplate seal and a plate roller. The same cycling conditions were used for both instruments: 95 °C for 10 min, followed by 6 cycles of [95 °C for 10 s, 61 °C for 15 s, 68 °C for 60 s], followed by 54 cycles of [95 °C for 10 s, 64 °C for 15 s, 68 °C for 30 s].

### Sequencing comparison for cancer cell line mixtures

Targeted PCR primers were designed using IDT’s rhAmpSeq design tool for all 32 cancer cell line variants. The same samples that were profiled using digital PCR were also profiled through sequencing. Amplicons were generated using the rhAmpSeq for CRISPR library preparation protocol per the manufacturer’s instructions and sequenced on an Illumina MiniSeq (2 × 150 bp). Raw sequencing data was converted to fastq using GATK IlluminaBasecallsToFastq, and then aligned to hg38 using bwa mem^29^. Genome coordinates for variants of interest were then used to calculate pileups using GATK DepthOfCoverage^30^.

### Probe-based qPCR assay upgraded to USE-PCR

A set of 9 primer systems and 9 target-specific probes were designed to detect common tick-borne pathogens (Table S11). Control qPCR reactions with synthetic templates were performed as previously described^31^. The TBP qPCR primers along with the RNAse P control assay were modified to incorporate RNAse H chemistry at the 3’ end and USE-PCR signal encoding tags at the 5’ end (Table S12). Primers were synthesized by IDT as described above. A 20X SNP detection primer mix was made by combining all 10 tag containing primers at 1 μM each and the 10 non-tag containing primers at 6 μM each. A 45X reference dye surfactant mix was prepared with a final concentration of 6.75 μM unquenched Cy5.5 probe (poly(T)10) along with 8% Tween-20. Reactions were prepared with 10 μL rhAmp Genotyping Mastermix, 1 μL 20X primer mix, 2 μL 40X universal probe mix, 0.4 μL Cy5.5 probe and Tween mix and thermal cycled on the QIAcuity according to the following protocol: 95 °C for 10 min, followed by 6 cycles of [95 °C for 15 s, 60 °C for 30 s, 72 °C for 120 s] and 56 cycles of [95 °C for 15 s, 64 °C for 30 s, 72 °C for 60 s].

### Statistical analysis

The ternary encoding scheme with universal probes creates four possible partition signal amplitude levels in each channel: 0 (no tags were present), 1 (only tags with the “1i” low-concentration universal probe were present), 2 (only tags with the “2i” high-concentration universal probe were present), or 3 (tags with both “1i” and “2i” universal probes were present). Amplitude thresholds were used to divide partitions between these four levels in each channel, and the levels across all four channels were used to create an overall label for that partition. For example, a partition with measured signal 2220 has amplitude level 2 in the first three color channels and amplitude level 0 in the fourth. For this analysis, thresholds were set for each instrument by manual inspection of a representative sample with all targets present, and then were kept constant across all samples, wells, and runs on that instrument.

One challenge with this encoding scheme is that partitions may generate a signal that is ambiguous as to which tags were present in that partition. For example, if three tags have labels 1000, 0100, and 1100, a partition with an observed label of 1100 could have one of five different tag combinations present: 1100, 1000 + 0100, 1100 + 1000, 1100 + 0100, or 1100 + 1000 + 0100. In order to calculate a call for the number of copies of each tag present in the original sample, we compute the relative probabilities of these various possible target compositions. We assume that Poisson statistics are applicable, i.e. that all DNA molecules in the original sample are distributed randomly, uniformly, and independently between the dPCR partitions. The analysis procedure is summarized below for a 4-channel encoding scheme, but the same logic applies for any universal tag multi-spectral encoding scheme across any number of color channels.

1. Every partition is assigned an amplitude label in each channel based on comparison to predefined amplitude thresholds for each channel.
2. Every partition with a label of 0000 is known to contain no amplified targets, so the number of 0000 partitions gives us our number of null partitions, or *P*_0000_*.*
3. Partitions with a label corresponding to a target with a probe in only one color channel (e.g. 1000 or 0100) are known to contain exactly that single target and no others. Based on the ratio between the number of these single-channel partitions and the null count, we can compute how many copies of the corresponding single-channel targets must be present using Poisson statistics. For example, given the count *P*_1000_ of partitions with label 1000, we can calculate the number of copies per partition of the 1000 target as
  3 $$\lambda_{{{1}000}} = {\text{ln}}({1} + P_{{{1}000}} /P_{0000} )$$
  Assuming there are *N* total partitions on the plate, this gives us a total copy number for the 1000 target of *N*λ_1000_.
4. Next, we consider partitions with a label corresponding to a target with a probe in exactly two color channels, e.g. 1100. As mentioned above, such partitions may contain various different target combinations. However, due to step 3 we know the concentration of all possible single-channel component targets (in this case, 1000 and 0100). We first calculate a number of “expected counts”, or the number of partitions we would expect to be at 1100 due to the 1000 + 0100 combination if no other targets were present:
  4 $$E_{{{11}00}} = P_{0000} \cdot [P_{{{1}000}} /P_{0000} ] \cdot [P_{{0{1}00}} /P_{0000} ]$$
  This gives us an initial corrected count
  5 $$P_{1100}^{\prime } = P_{{{11}00}} - E_{{{11}00}}$$
  representing the expected number of 1100 partitions that actually contain the 1100 target.
5. Even if a 1100 partition has the 1100 target present, it may also have copies of the 1000 and/or 0100 targets present. To correct for this, we need to multiply by a factor of *e*^−λ*X*^ for each of the other targets *X* that may be present in our partitions of interest. This gives us a final corrected count of
  6 $${P}_{1100}^{*}={P}_{1100}^{\prime} {{e}^{-{\lambda }_{1000}}{e}^{-{\lambda }_{0100}} }$$
  which represents the expected number of partitions containing only the 1100 target and no others. This allows us to calculate
  7 $$\lambda_{{{11}00}} = {\text{ ln}}({1} + [P_{1100}^{*} /P_{0000} ])$$
6. Once steps 4 and 5 have been performed for all targets with 2 channel tags, we can repeat this for all targets with 3 channel tags, and then all targets with 4 channel tags, etc. At the end of this process, we have successfully resolved ambiguities to compute how many copies of each target were present in the original sample.

### Advantage of multi-spectral encoding

In applications where one is interested in the total number of counts across multiple targets, the level of accuracy can be increased by increasing the number of targets in order to decrease sampling variance^5^. In classic multiplexing methods where each target generates signal in a separate channel, one can calculate each target count independently, so sampling variance goes down by a factor of $\sqrt{n}$ for *n* multiplexed targets. However, in the universal signal encoding scheme the inherent partition ambiguity means that targets cannot be analyzed independently, so the $\sqrt{n}$ variance scaling may not apply.

To determine the sampling variance, we conducted Monte Carlo simulations of a dPCR run with 4 channels and 26,000 partitions using a 1-channel-per-target 4-plex and the universal signal encoding scheme for a 32-plex. In each run the expected number of copies was the same for all targets, but the actual number of copies present was set by independent Poisson sampling for each target. We assume that all targets in all partitions are fully amplified and that all partitions were correctly classified into an amplitude label based on the targets present. The number of copies of each target was then calculated using classic Poisson analysis (for the 4-plex) or the analysis scheme described above (for the 32-plex), and the sum of copies across all targets was stored. Simulations were run at expected copy numbers per tag ranging from 1 to 5000, and at each concentration the total copy error level was calculated as the coefficient of variation (CV) over 10,000 runs. Results are shown in Fig. S13.

We can see that at lower copy numbers, the 32-plex has a significantly lower error level than the 4-plex by a factor of about $\sqrt{8}$. This makes sense, as at such low concentrations there are very few partitions with multiple targets, so the 32-plex analysis is effectively independent and the traditional $\sqrt{n}$ scaling should hold. As target concentration increases the oversaturation error due to multi-target presence starts to contribute significantly in the 32-plex case, leading to a crossover point at roughly 1000 copies per target after which the 4-plex yields better results. For our application we generally expect sample concentrations to be significantly below 1000 copies/target, allowing the 32-plex scheme to yield significant accuracy improvements.

## Electronic supplementary material

Below is the link to the electronic supplementary material.

Supplementary Material 1

Supplementary Material 2

Supplementary Material 3

## Data Availability

Targeted amplicon sequencing data for the cancer cell line experiments has been deposited in the Short Read Archive (SRA) under BioProject PRJNA1233339. All other data are available in the main text or the supplementary materials. Raw instrument digital PCR data is available from the corresponding author upon reasonable request.
