# Supplementary material for: Multiplexed, universal probe-based rare variant detection with USE-PCR
Source: Sci Rep. 2025 Jul 4;15:23947. doi: 10.1038/s41598-025-08814-5 (PMC12227673; doi:10.1038/s41598-025-08814-5)
Supplement: Supplementary file 1 — Supplementary Material 1 [file 41598_2025_8814_MOESM1_ESM.docx]

Supplementary Materials for

Title

Multiplexed, universal probe-based rare variant detection with USE-PCR

John Alvarado *et al*.

Corresponding author, [jschwartz@chromacode.com](mailto:jschwartz@chromacode.com)

**This PDF file includes:**

Figs. S1 to S13

Tables S1 to S12

**Other Supplementary Materials for this manuscript include the following:**

USE-PCR_supplementary_tables_v2.xlsx


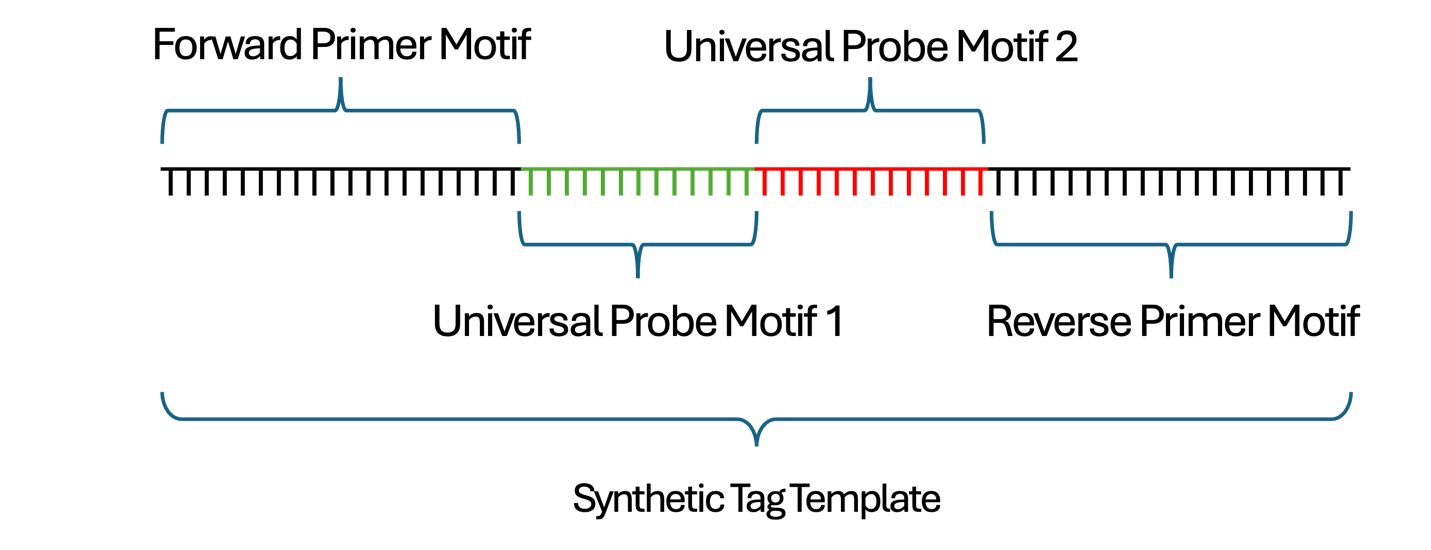


Figure S1. Synthetic tag template architecture. Common forward and reverse primers based on ACTB were designed to flank all 32 unique tag sequences. Each tag sequence is comprised of one or two universal probe motifs. Example sequences are provided in Table S2.


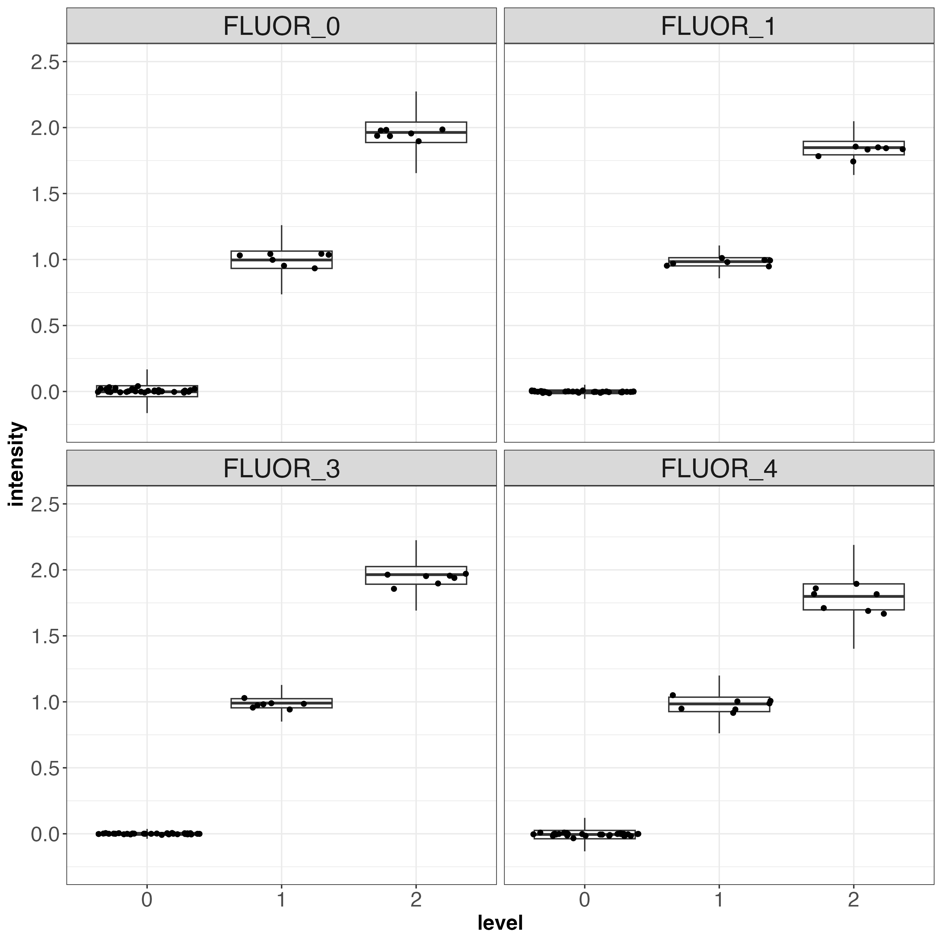


**Figure S2**. **Singleplex testing of 32 individual synthetic tags measured on the QIAGEN QIAcuity.** Each panel represents the intensity distribution of partitions for a specific tag, as defined in **Table S1**. The y-axis shows the normalized intensity values detected across partitions, with boxplots indicating the distribution: the box spans the interquartile range (IQR), the horizontal line indicates the median, and whiskers extend to 1.5 times the IQR. Mean intensity values for each tag are depicted by black circles. Outliers are not displayed due to the high partition count (>500,000 per panel), but they are included in the calculation of medians and IQRs.


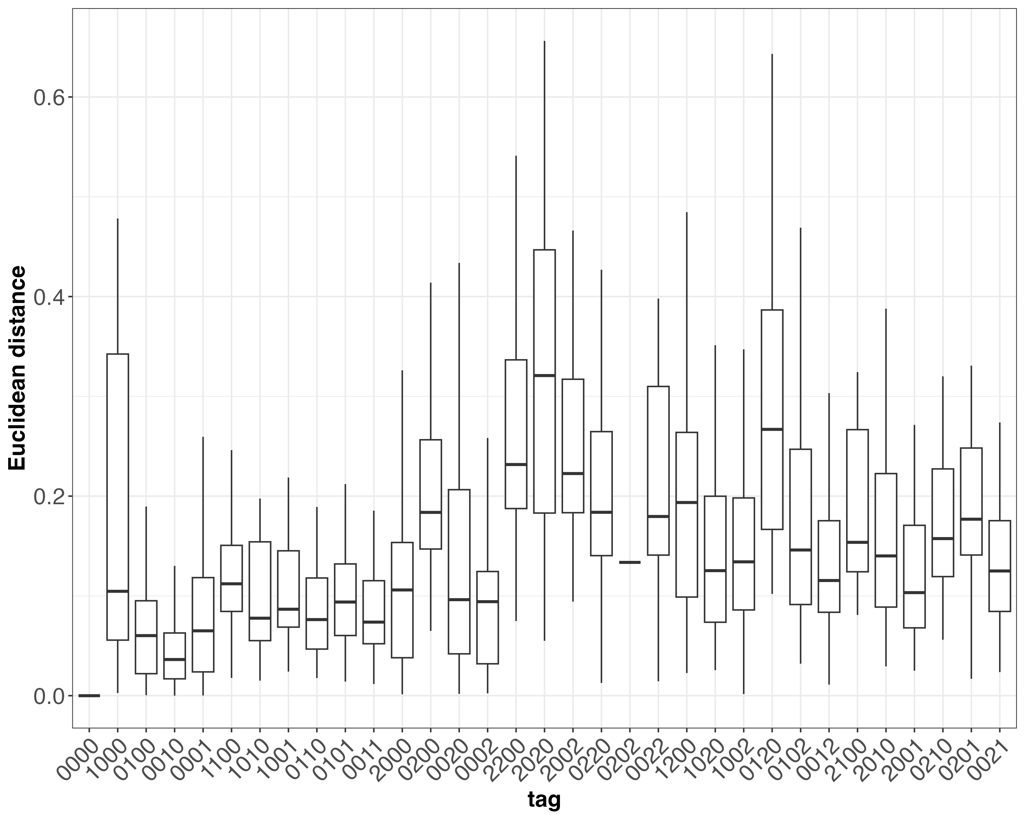


**Figure S3**. **Euclidean distance between experimental signal and target signal coordinates.** A synthetic tag mixture containing 32 synthetic tags was measured on the QIAGEN QIAcuity. The Euclidean distance between the signal from each partition and the target tag signal location was calculated in the 4 color dimensions. Each box extends from the first to third quartile; the line is the median; the whiskers extend to indicate variability outside Q1 and Q3. Outliers are not displayed due to the high partition count, but they are included in the calculation of medians and IQRs.


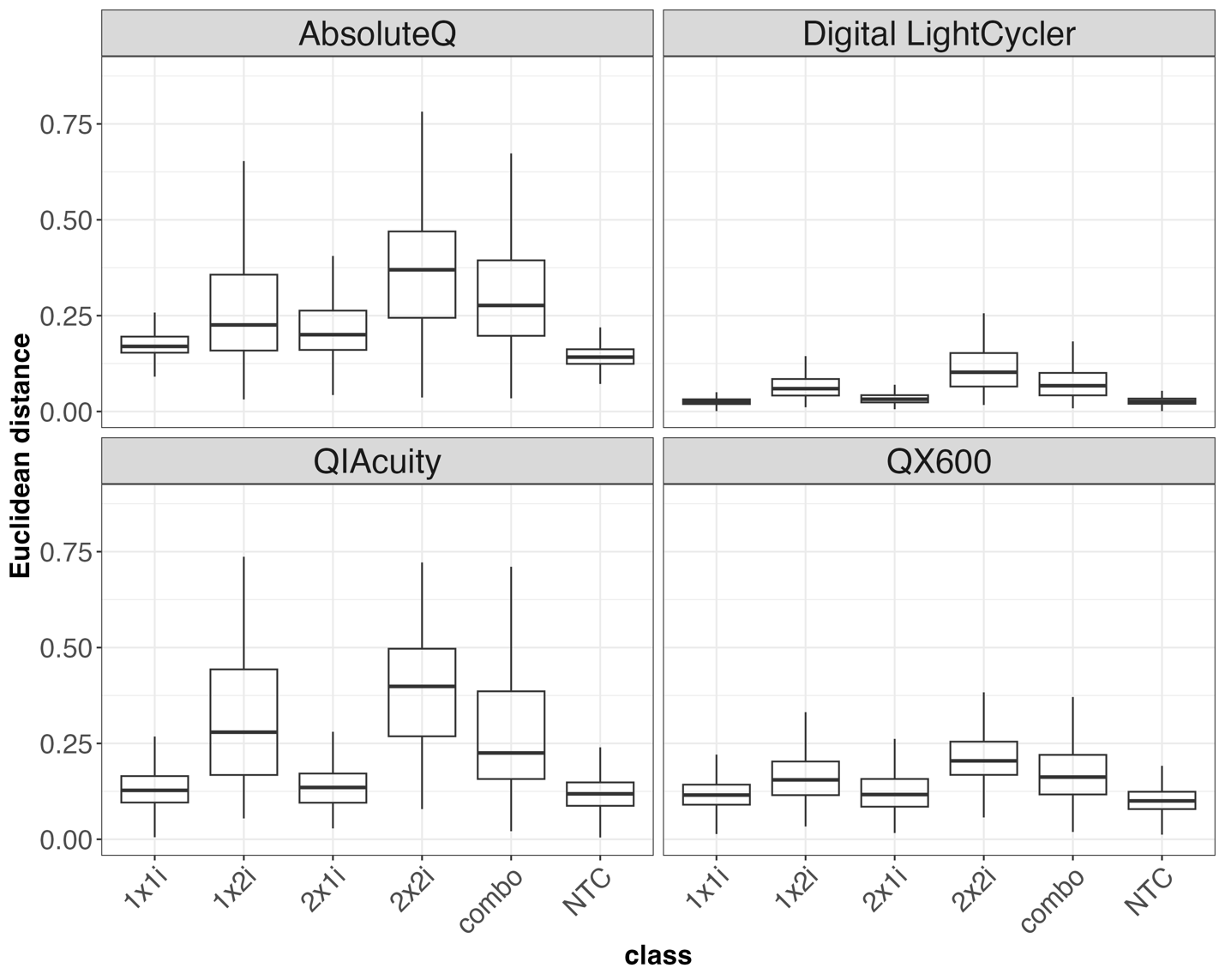


**Figure S4. Euclidean distance variation across platforms.** A synthetic tag mixture containing all 32 synthetic tags was measured on all four instrument platforms. The Euclidean distance between the signal from each partition and the target tag signal location was calculated in the 4 color dimensions. Tags were then grouped according to their composition of 1i and/or 2i signaling probes. Each box extends from the first to third quartile; the line is the median; the whiskers extend to indicate variability outside Q1 and Q3. Outliers are not displayed due to the high partition count, but they are included in the calculation of medians and IQRs.


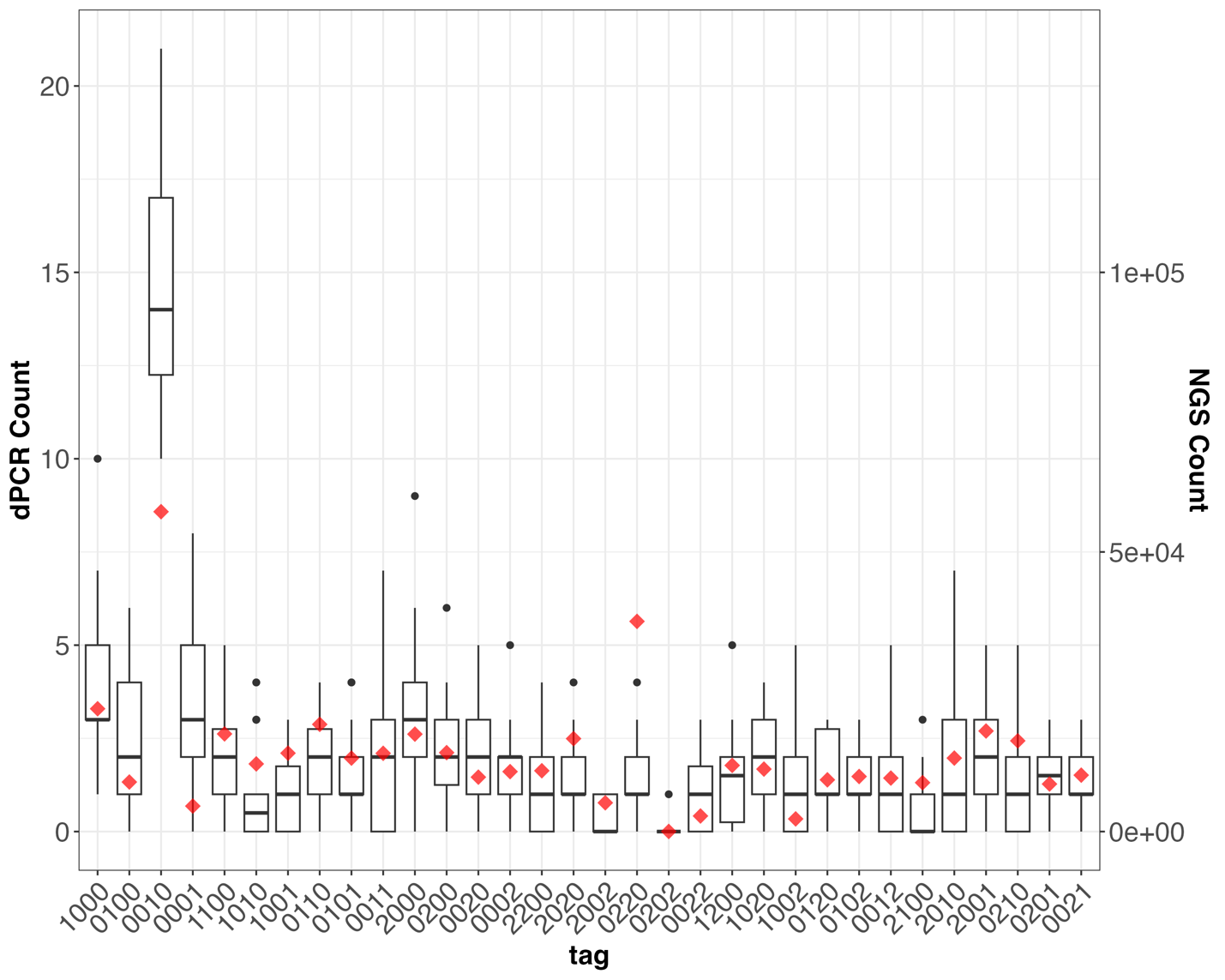


**Figure S5.** **Synthetic tag detection and resolution at low copy on the QIAGEN QIAcuity.** A mixture containing 32 synthetic tag templates was diluted to 0.03% with respect to a reference tag kept at a constant copy number on the QIAcuity. The signal from each partition was assigned to a corresponding tag identity per the methods section; total counts for each tag are given on the y-axis. The box extends from the first to third quartile; the line is the median; the whiskers extend to indicate variability outside Q1 and Q3. Sequencing the same sample gave the corresponding NGS reads on the right y-axis.


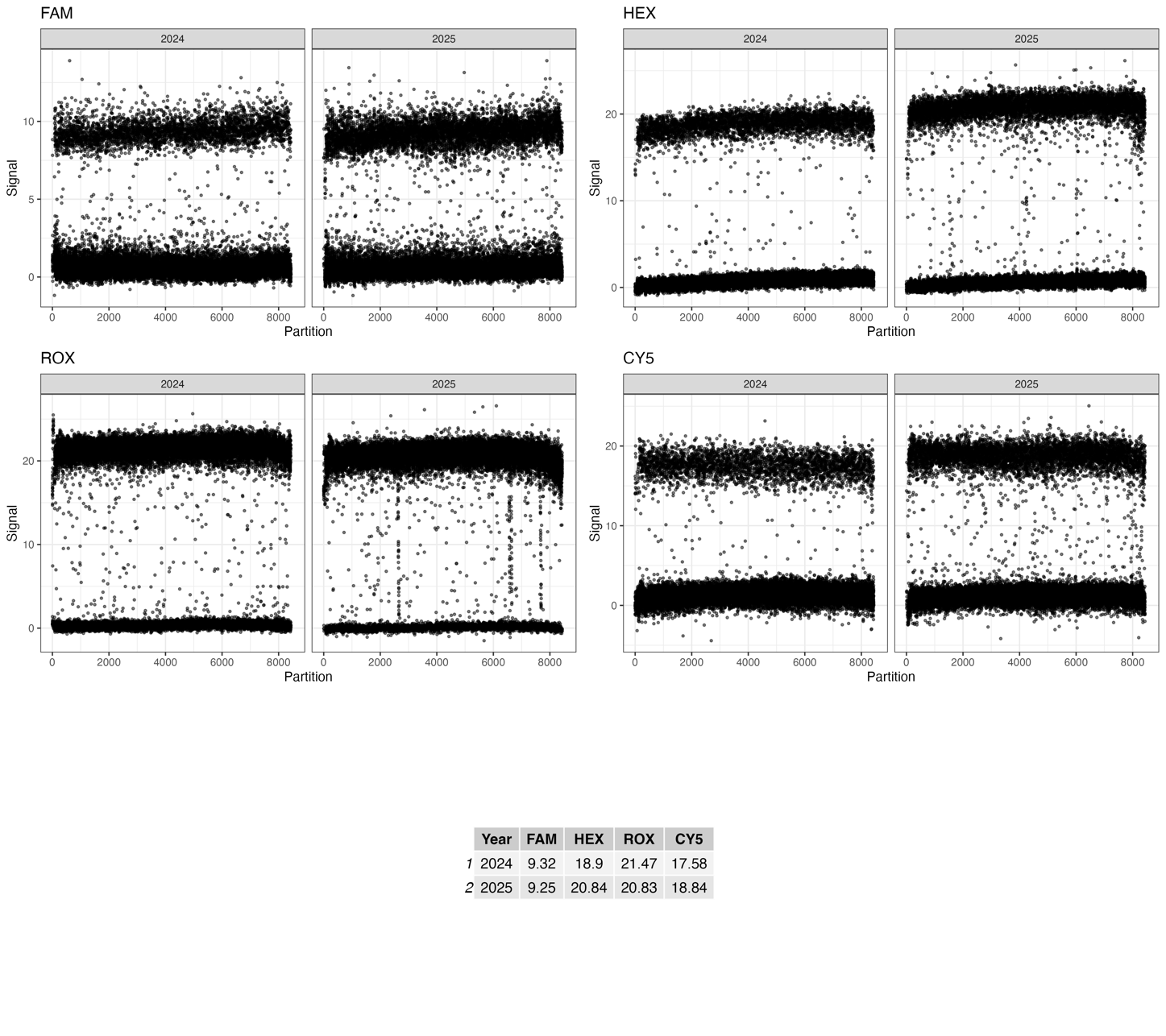
**Figure S6**. **Universal probes are stable over one year of storage.** A single lot of universal probe mix was tested with synthetic tag templates (tags 1000, 0100, 0010, and 0001) in May 2024 and May 2025 on the QIAGEN QIAcuity (8.5k plate). Intensity values for each of four color channels are shown, with N=3 replicates overlayed on each plot. The table below the figure shows the median intensity values of the positive partition cloud for each year and color channel.


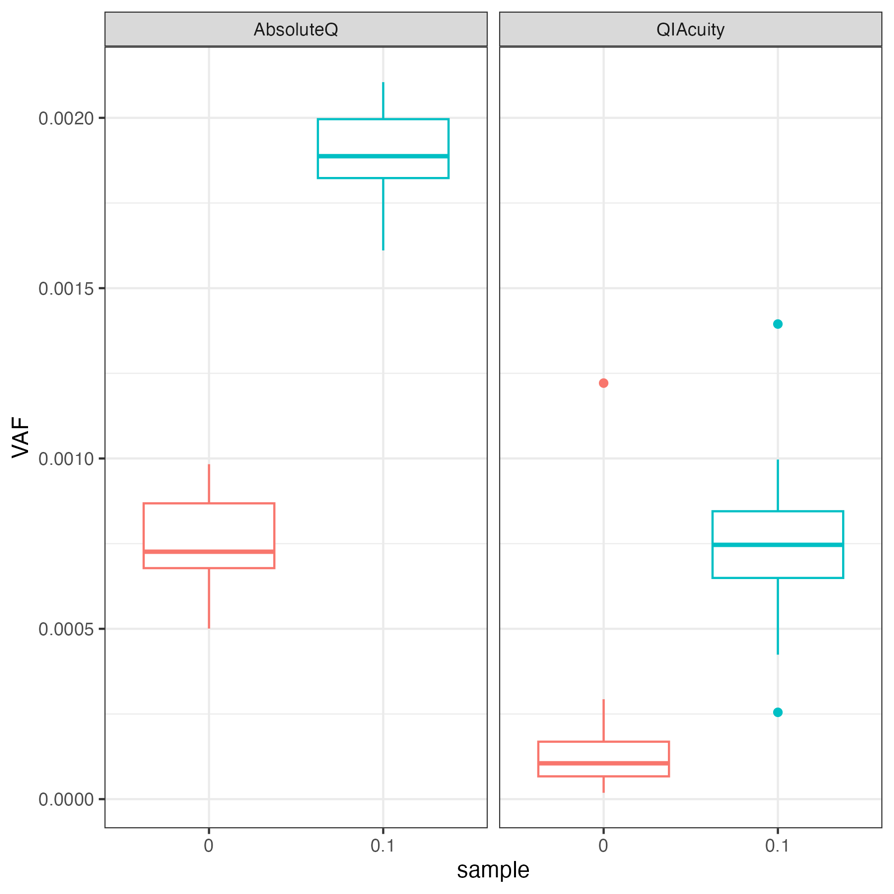


**Figure S7** – **USE-PCR enables accurate rare SNV detection across dPCR platforms.** A 0.1% relative fraction SNV sample and a 0% background genomic DNA sample were processed using a common primer mix and universal probe mix on the AbsoluteQ and QIAcuity. The mean variant allele frequency across all 32 SNV synthetic targets was determined for N=21 replicates **(**0.1% relative fraction sample on the AbsoluteQ) and N=24 replicates (all other samples).


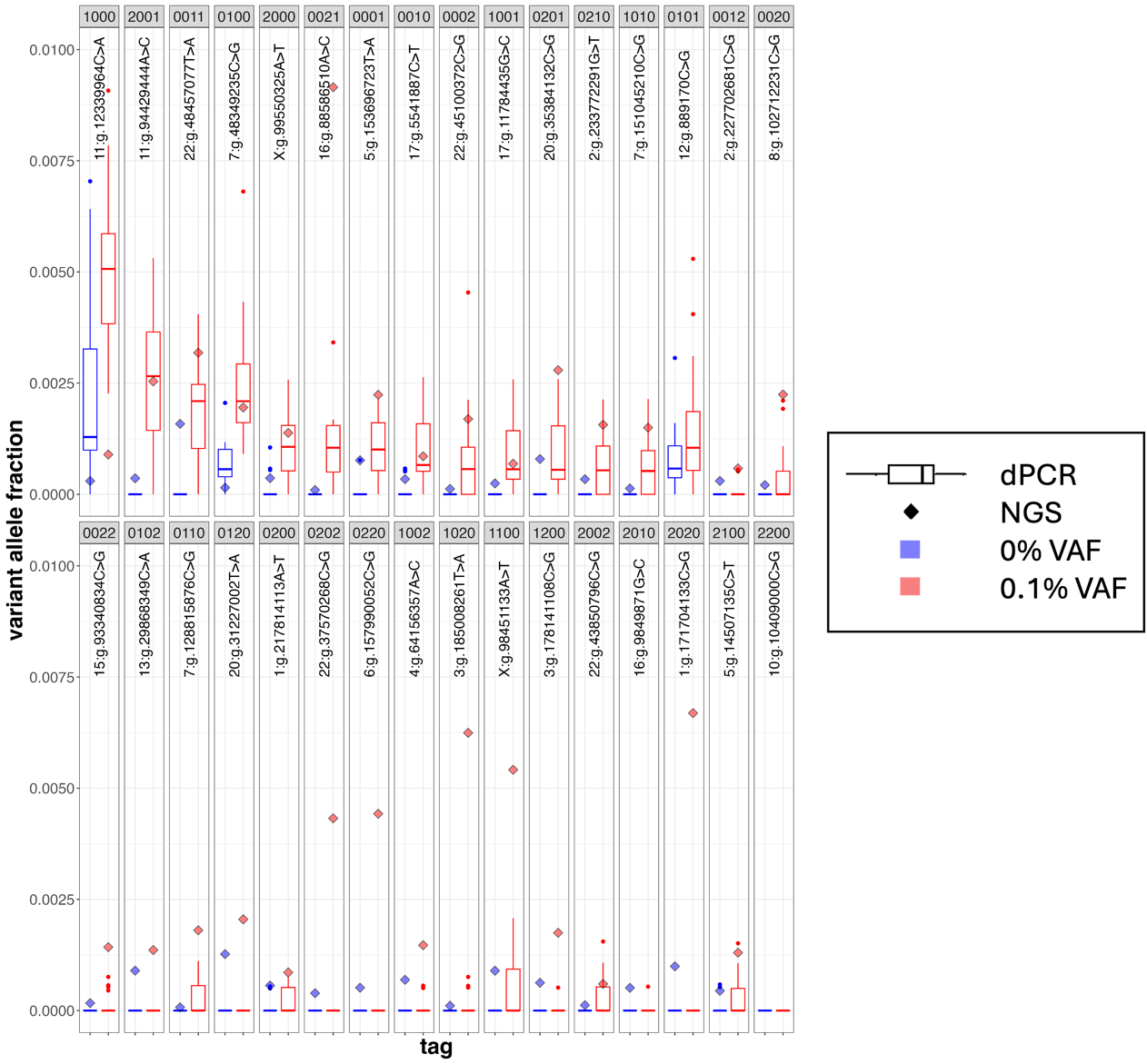


**Figure S8. 32-plex USE-PCR SNV assay with synthetic templates on QIAcuity.** A mixture of synthetic, SNV-containing templates was prepared and mixed at 0.1% dilution with a background cell line (HCC1395BL). A total of 24 0% VAF replicates and 24 0.1% VAF replicates were processed on the QIAcuity. The variant allele fraction of each variant was determined with respect to a reference amplicon in the background cell line. Partitions were assigned to tags based on universal signal deconvolution (Methods section). Sequencing reads for both samples was performed in parallel (diamonds) and the variant allele fraction was determined with respect to the wild type allele.


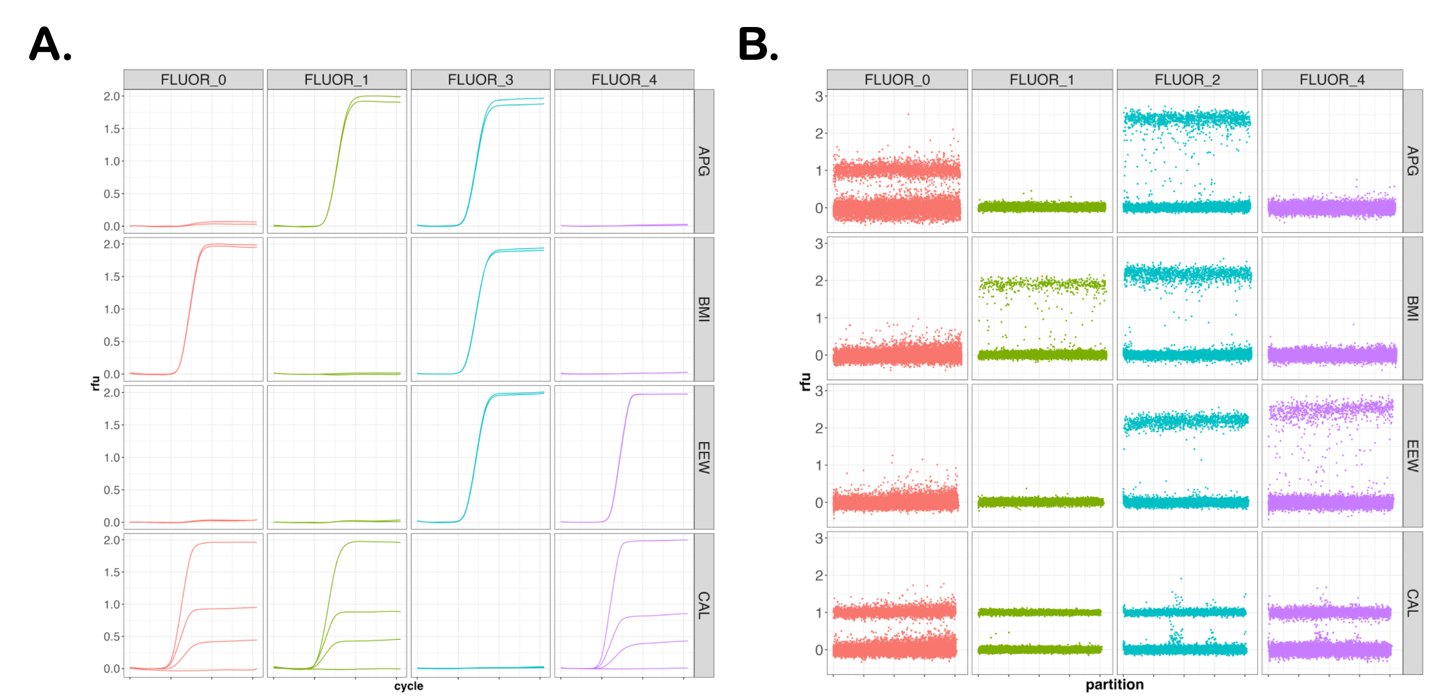


**Figure S9. USE-PCR enables assay portability from qPCR to dPCR.**  A) Panels show a qPCR probe-based assay for tick-borne pathogens. The top three rows illustrate multispectral encoding for *anaplasma phagocytophilum* (APG), *babesia microti* (BMI), and *ehrlichia ewingii* (EEW) each with an internal extraction control in FLUOR_3, and the bottom row is a calibrator target (CAL). B) The same targets were re-encoded with USE-PCR as a 1000 (APG), 0200 (BMI), 0002 (EEW), with an internal sample input control encoded at 0020.


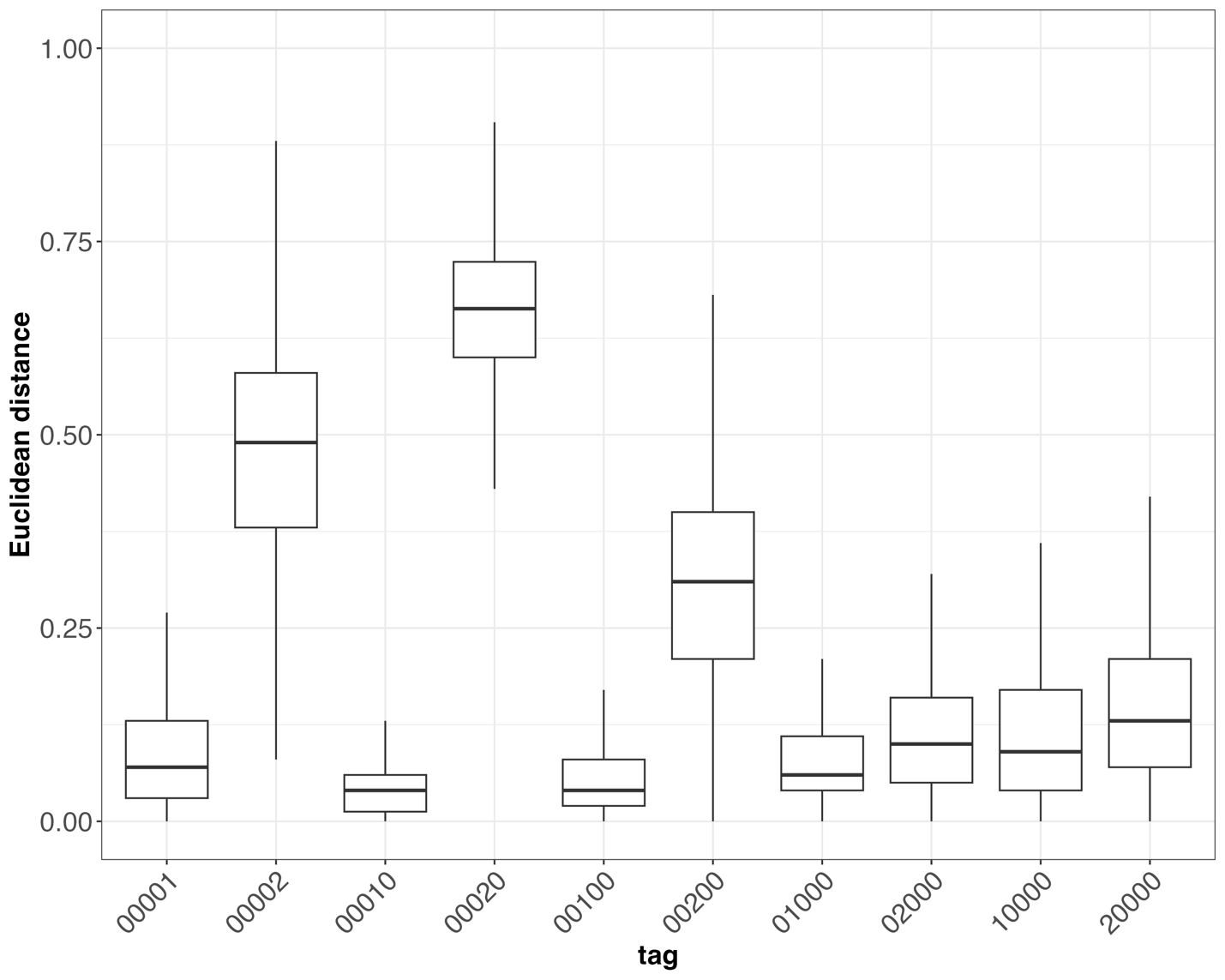


**Figure S10**. **Tick borne pathogen panel was measured on the QIAGEN QIAcuity.** The Euclidean distance between the signal from each partition and the target tag signal location was calculated in the 5 color dimensions. Each box extends from the first to third quartile; the line is the median; the whiskers extend to indicate variability outside Q1 and Q3. Outliers are not displayed due to the high partition count, but they are included in the calculation of medians and IQRs.


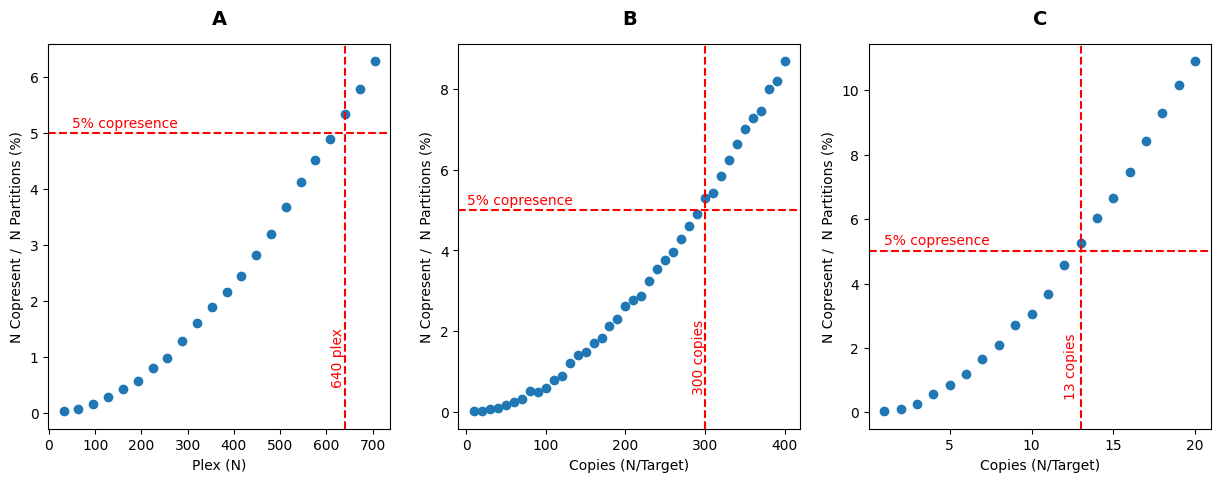


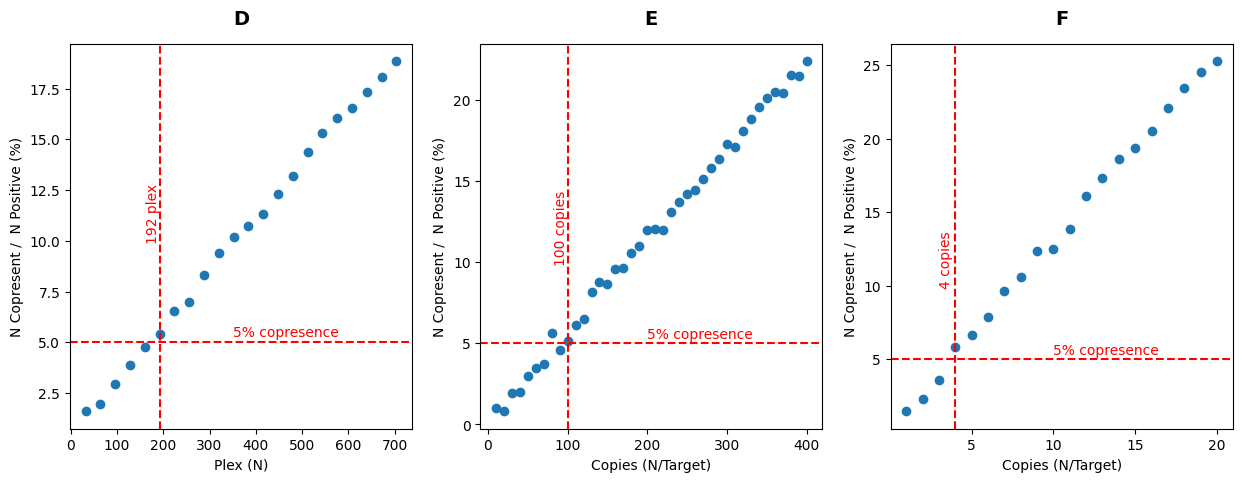


**Figure S11**. **Poisson simulations illustrating the relationship between target concentration, panel size, and signal co-presence in USE-PCR.** Three simulation scenarios were run assuming 26,000 partitions and random Poisson distribution of molecules across partitions: (A) At a fixed target concentration of 15 copies/target, the number of targets was varied to determine the maximum multiplexing level where the fraction of total partitions containing 2 or more molecules (“co-present”) surpasses 5%. (B) At a fixed panel size of 32 targets, the number of copies per target was increased to determine the concentration limit where the fraction of total partitions containing 2 or more molecules starts to exceed 5%. (C) For a high-plex assay (728 targets), the number of copies per target was varied to determine the concentration threshold where the fraction of total partitions that exhibit co-presence exceeds 5%. D-F) Same simulations as A-C), with the y-axis adjusted to reflect the fraction of positive partitions that exhibit copresence. These results provide practical guidance on balancing multiplexing depth with target abundance to maintain minimal signal overlap within partitions.


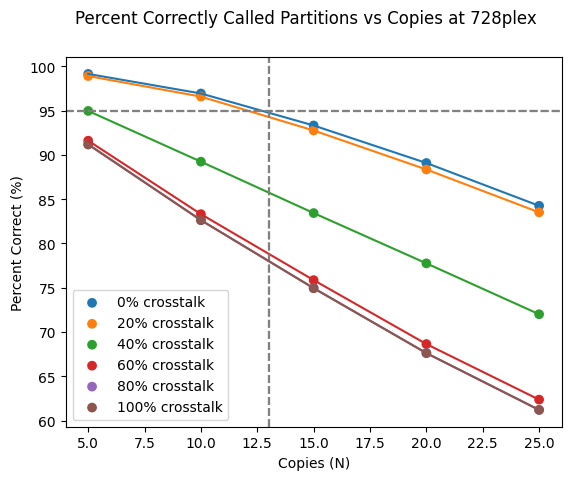


**Figure S12.** **Partition call accuracy declines with increasing target abundance under high-plex conditions and fixed spectral crosstalk.** Simulated 728-plex reactions (26,000 partitions, 6 detection channels) were analyzed across a range of per-target copy numbers. The amount of crosstalk from ch3 into ch4 was varied to understand the relationship of calling accuracy (ie. percent correctly called partitions) to crosstalk and input copies. Using a model system of constant but representative target cloud distributions we show at low crosstalk, copresence effects dominate calling accuracy decreases (ie. by increasing input copies). At high crosstalk levels this analysis shows the need to inverse crosstalk in software to maintain high calling accuracy.


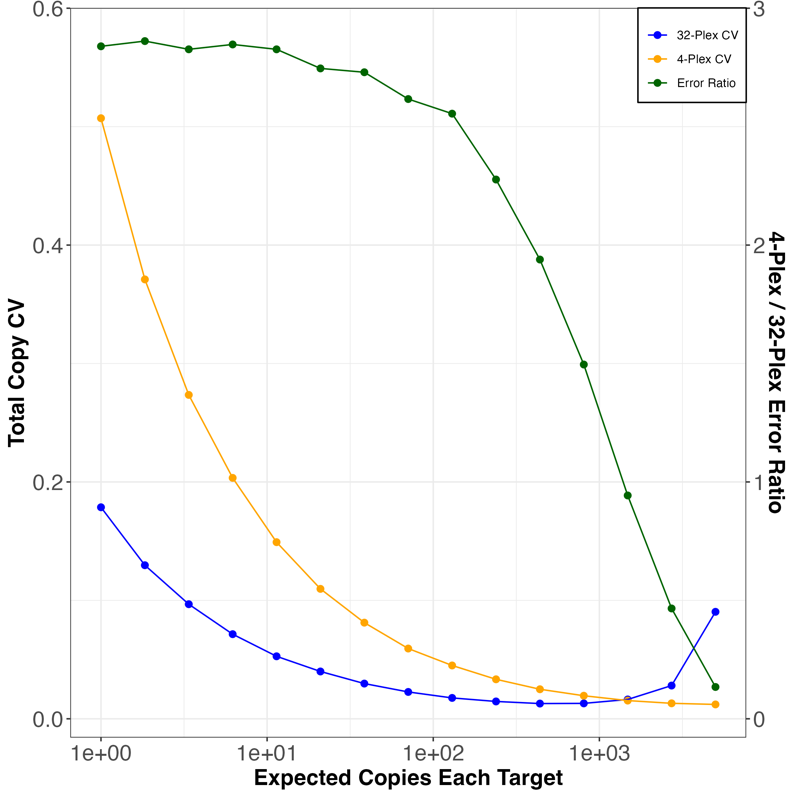


Figure S13. Monte Carlo simulation of Total Copy CV and Error Ratio for a standard 4-plex and USE-PCR for a 32-plex. The number of copies of each target was calculated using classic Poisson analysis (for the 4-plex) or the analysis scheme described in Methods (for the 32-plex), and the sum of copies across all targets was stored. Simulations were run at expected copy numbers per tag ranging from 1 to 5000, and at each concentration the total copy error level was calculated as the coefficient of variation (CV) over 10000 runs.

Table S1. Signal encoding scheme for color-coded tags on a four color channel instrument. 1 = probe binding site for a universal probe at a “1i” intensity level; 2 = probe binding site for a universal probe at a “2i” intensity level.

|  | FLUOR_1 | FLUOR_2 | FLUOR_3 | FLUOR_4 |
| --- | --- | --- | --- | --- |
| tag_1000 | 1 | 0 | 0 | 0 |
| tag_0100 | 0 | 1 | 0 | 0 |
| tag_0010 | 0 | 0 | 1 | 0 |
| tag_0001 | 0 | 0 | 0 | 1 |
| tag_1100 | 1 | 1 | 0 | 0 |
| tag_1010 | 1 | 0 | 1 | 0 |
| tag_1001 | 1 | 0 | 0 | 1 |
| tag_0110 | 0 | 1 | 1 | 0 |
| tag_0101 | 0 | 1 | 0 | 1 |
| tag_0011 | 0 | 0 | 1 | 1 |
| tag_2000 | 2 | 0 | 0 | 0 |
| tag_0200 | 0 | 2 | 0 | 0 |
| tag_0020 | 0 | 0 | 2 | 0 |
| tag_0002 | 0 | 0 | 0 | 2 |
| tag_2200 | 2 | 2 | 0 | 0 |
| tag_2020 | 2 | 0 | 2 | 0 |
| tag_2002 | 2 | 0 | 0 | 2 |
| tag_0220 | 0 | 2 | 2 | 0 |
| tag_0202 | 0 | 2 | 0 | 2 |
| tag_0022 | 0 | 0 | 2 | 2 |
| tag_1200 | 1 | 2 | 0 | 0 |
| tag_1020 | 1 | 0 | 2 | 0 |
| tag_1002 | 1 | 0 | 0 | 2 |
| tag_0120 | 0 | 1 | 2 | 0 |
| tag_0102 | 0 | 1 | 0 | 2 |
| tag_2100 | 2 | 1 | 0 | 0 |
| tag_0012 | 0 | 0 | 1 | 2 |
| tag_2010 | 2 | 0 | 1 | 0 |
| tag_0210 | 0 | 2 | 1 | 0 |
| tag_2001 | 2 | 0 | 0 | 1 |
| tag_0201 | 0 | 2 | 0 | 1 |
| tag_0021 | 0 | 0 | 2 | 1 |

Table S2. Example construction of synthetic color-coded tag sequences. Here, “tag” refers to the unique combination of probe-binding sites, based on colors and amplitude levels, for the hydrolysis probes in the tag. For example, the tag “0102” on the AbsoluteQ encodes a 1i signal in VIC and a 2i signal in Cy5. Sequences are listed in 5’ to 3’ orientation.

| tag | univ_forward_site | padding | tag_1 | padding | tag_2 | univ_reverse_site |
| --- | --- | --- | --- | --- | --- | --- |
| "0001" | CCTTGCACATGCCGGAG | AT | probe_1 | AT |  | CAAAGGCGAGGCTCTGT |
| "0002" | CCTTGCACATGCCGGAG | AT | probe_2 | AT |  | CAAAGGCGAGGCTCTGT |
| "0010" | CCTTGCACATGCCGGAG | AT | probe_3 | AT |  | CAAAGGCGAGGCTCTGT |
| "0011" | CCTTGCACATGCCGGAG | AT | probe_3 | AT | probe_1 | CAAAGGCGAGGCTCTGT |
| "0012" | CCTTGCACATGCCGGAG | AT | probe_3 | AT | probe_2 | CAAAGGCGAGGCTCTGT |
| "0020" | CCTTGCACATGCCGGAG | AT | probe_4 | AT |  | CAAAGGCGAGGCTCTGT |
| "0021" | CCTTGCACATGCCGGAG | AT | probe_4 | AT | probe_1 | CAAAGGCGAGGCTCTGT |
| "0022" | CCTTGCACATGCCGGAG | AT | probe_4 | AT | probe_2 | CAAAGGCGAGGCTCTGT |
| "0100" | CCTTGCACATGCCGGAG | AT | probe_5 | AT |  | CAAAGGCGAGGCTCTGT |
| "0101" | CCTTGCACATGCCGGAG | AT | probe_5 | AT | probe_1 | CAAAGGCGAGGCTCTGT |
| "0102" | CCTTGCACATGCCGGAG | AT | probe_5 | AT | probe_3 | CAAAGGCGAGGCTCTGT |
| "0110" | CCTTGCACATGCCGGAG | AT | probe_5 | AT | probe_2 | CAAAGGCGAGGCTCTGT |
| "0120" | CCTTGCACATGCCGGAG | AT | probe_5 | AT | probe_4 | CAAAGGCGAGGCTCTGT |
| "0200" | CCTTGCACATGCCGGAG | AT | probe_6 | AT |  | CAAAGGCGAGGCTCTGT |
| "0201" | CCTTGCACATGCCGGAG | AT | probe_6 | AT | probe_1 | CAAAGGCGAGGCTCTGT |
| "0202" | CCTTGCACATGCCGGAG | AT | probe_6 | AT | probe_2 | CAAAGGCGAGGCTCTGT |
| "0210" | CCTTGCACATGCCGGAG | AT | probe_6 | AT | probe_3 | CAAAGGCGAGGCTCTGT |
| "0220" | CCTTGCACATGCCGGAG | AT | probe_6 | AT | probe_4 | CAAAGGCGAGGCTCTGT |

**Table S3.** **Universal probe mix formulations were tailored to each instrument’s filter sets and detection capabilities.** For each instrument, two probes were designed and synthesized for each of four different color channels. The probes were then assembled into a platform-specific universal probe mix. The final probe concentrations in the dPCR reaction well are shown.

|  | FAM | HEX | TAMRA | ROX | CY5 |
| --- | --- | --- | --- | --- | --- |
| Absolute Q | Probe 1: 25 nM  Probe 2: 50 nM | Probe 1: 50 nM  Probe 2: 100 nM | Probe 1: 50 nM  Probe 2: 100 nM | - | Probe 1: 50 nM  Probe 2: 100 nM |
| Digital LightCycler | Probe 1: 25 nM  Probe 2: 50 nM | Probe 1: 25 nM  Probe 2: 50 nM | - | Probe 1: 25 nM  Probe 2: 50 nM | Probe 1: 25 nM  Probe 2: 50 nM |
| QIAcuity  4-channel mix | Probe 1: 25 nM  Probe 2: 50 nM | Probe 1: 50 nM  Probe 2: 100 nM | - | Probe 1: 50 nM  Probe 2: 100 nM | Probe 1: 50 nM  Probe 2: 100 nM |
| QX600 | Probe 1: 25 nM  Probe 2: 50 nM | Probe 1: 25 nM  Probe 2: 50 nM | - | Probe 1: 25 nM  Probe 2: 50 nM | Probe 1: 25 nM  Probe 2: 50 nM |
| QIAcuity 5-channel mix | Probe 1: 12.5 nM  Probe 2: 25 nM | Probe 1: 25 nM  Probe 2: 50 nM | Probe 1: 25 nM  Probe 2: 50 nM | Probe 1: 25 nM  Probe 2: 50 nM | Probe 1: 25 nM  Probe 2: 50 nM |

**Table S4A.** **Synthetic tag reaction setup and thermal cycling conditions for each instrument platform.**

|  | Absolute Q | Digital LightCycler | QIAcuity | QX600 |
| --- | --- | --- | --- | --- |
| 20X Primer Mix (6 μM forward/reverse) | 0.6 μL | 2 μL | 2.25 μL | 1 μL |
| Synthetic Tag Template Mix | 5 μL | 5 μL | 5 μL | 5 μL |
| Universal Probe Mix (20X) | 0.6 μL | 2 μL | 2.25 μL | 1 μL |
| Platform-specific Master Mix (below) | 2.4 μL | 8 μL | 11.25 μL | 5 μL |
| 1X TE | 3.4 μL | 23 μL | 24.25 μL | 9 μL |
| Total volume | 12 μL | 40 μL | 45 μL | 20 μL |
|  |  |  |  |  |
| Thermal cycling |  |  |  |  |
| Activation | 95°C, 10 min | 95°C, 2 min | 95°C, 2 min | 95°C, 10 min |
| Cycling | [95°C, 20 sec then 58°C, 120 sec] x 40 cycles | | | |
| Deactivation |  |  |  | 98°C, 10 min |

**Table S4B.** **PCR MasterMixes for each instrument.**

| Manufacturer | Instrument | Catalog Number | Name |
| --- | --- | --- | --- |
| Thermo Fisher | Thermo Fisher | A52490 | Absolute Q™ DNA Digital PCR Master Mix (5X) |
| QIAGEN | QIAcuity | 1133251 | QIAcuity MasterMix |
| BioRad | QX600 | 1863024 | ddPCR™ Supermix for Probes (No dUTP) |
| Roche | DLC | 09393544001 | Digital LightCycler®  5x DNA Master |

Table S5. Tag classification accuracy using individual synthetic templates on the QIAGEN QIAcuity, at both high and low copy number.

File: USE-PCR_supplementary_table_S5.xlsx

**Table S6. Digital PCR instrument specifications.** There is variation in sample loading volumes, interrogation volumes, and optimal dyes across four digital PCR platforms.

|  | Absolute Q | Digital LightCycler | QIAcuity | | QX600 |
| --- | --- | --- | --- | --- | --- |
| Setup Plate volume(μL) | 12 | 40 | 45** | 16* | 20 |
| Loaded into Array(μL) | 9 | 35 | 40 | 12 | 20 |
| Interrogated Volume | >95% | >85% | ~65% | ~25% | No ref |
| Partitions | 20000 | 28000 | 26000 | 8500 | 20000 |
| Format | 16 Well plate | 2x8 Well plate | 24 Well Plate | 96 Well Plate | 96 Well Plate |
| FAM | YES | YES | YES | YES | YES |
| HEX | YES | YES | YES | YES | YES |
| TAMRA | YES |  |  | YES |  |
| ROX |  | YES | YES | YES | YES |
| CY5 | YES | YES | YES | YES | YES |

*Used for SNV experiments

**Used for synthetic experiments

**Table S7**. **Statistical assessment of each SNV call in the 0.1 dilution sample with respect to background.** A mixture of synthetic variants from three cancer cell lines (HCC1395, HCC1187, HCC1143) were diluted in matched normal 1395BL. For the 0.1 dilution sample, the wilcox_sum_p_value was calculated for data generated on the AbsoluteQ vs. a 1395BL genomic DNA background. (@) Synthetic template not present. ( # ) Not statistically significant from genome DNA background.

|  |  | Wilcox_sum_p_value | |
| --- | --- | --- | --- |
| tag | chr_position_base_change | AbsoluteQ | QIAcuity |
| 1000 | 11:g.12339964C>A | 3.22e-7 | 5.96e-4 |
| 0100 | 7:g.48349235C>G | 7.93e-7 | 7.59e-8 |
| 0010 | 17:g.5541887C>T | 6.25e-7 | 1.61e-6 |
| 0001 | 5:g.153696723T>A | 1.47e-6 | 4.68e-8 |
| 1100 | X:g.98451133A>T | 3.27e-7 | 5.19e-4 |
| 1010 | 7:g.151045210C>G | 8.92e-9 | 9.18e-7 |
| 1001 | 17:g.11784435G>C | 5.19e-8 | 3.21e-7 |
| 0110 | 7:g.128815876C>G | 3.02e-6 | 2.31e-4 |
| 0101 | 12:g.889170C>G | 6.90e-3 @ | 6.56e-2 @ |
| 0011 | 22:g.48457077T>A | 2.24e-8 | 3.15e-9 |
| 2000 | X:g.99550325A>T | 9.91e-1 # | 3.55e-7 |
| 0200 | 1:g.217814113A>T | 5.26e-8 | 3.05e-2 |
| 0020 | 8:g.102712231C>G | 5.08e-10 | 2.42e-3 |
| 0002 | 22:g.45100372C>G | 2.73e-9 | 9.18e-7 |
| 2200 | 10:g.10409000C>G | 1.89e-2 |  |
| 2020 | 1:g.171704133C>G | 7.95e-10 |  |
| 2002 | 22:g.43850796C>G | 9.70e-5 | 1.13e-3 |
| 0220 | 6:g.157990052C>G | 1.97e-9 |  |
| 0202 | 22:g.37570268C>G | 2.06e-9 |  |
| 0022 | 15:g.93340834C>G | 4.98e-10 | 2.07e-2 |
| 1200 | 3:g.178141108C>G | 7.53e-9 @ | 3.38e-1 @ # |
| 1020 | 3:g.185008261T>A | 1.49e-8 | 2.07e-2 |
| 1002 | 4:g.64156357A>C | 1.49e-8 | 1.62e-1 # |
| 0120 | 20:g.31227002T>A | 9.17e-7 |  |
| 0102 | 13:g.29868349C>A | 9.82e-1 # |  |
| 0012 | 2:g.227702681C>G | 1.93e-9 | 8.11e-2 |
| 2100 | 5:g.14507135C>T | 1.74e-8 | 6.26e-2 |
| 2010 | 16:g.9849871G>C | 7.81e-10 | 3.38e-1 # |
| 2001 | 11:g.94429444A>C | 1.09e-8 | 8.81e-10 |
| 0210 | 2:g.233772291G>T | 4.99e-2 | 9.18e-7 |
| 0201 | 20:g.35384132C>G | 4.53e-9 | 3.21e-7 |
| 0021 | 16:g.88586510A>C | 2.55e-9 | 3.48e-8 |

 Table S8. USE-PCR partition calls for each of the 32 targets across three cancer cell lines at 12.5% dilution. A total of =3 replicates were run, and the partition counts represent total partitions across all three replicates on the QIAGEN QIAcuity platform. Targeted sequencing of each cell line was used to confirm which variants were present at a VAF >= 1% (colored shading).

| **tag** | **target** | **HCC1143** | **HCC1187** | **HCC1395** |
| --- | --- | --- | --- | --- |
| 1000 | 11:g.12339964C>A | 469 | 76 | 31 |
| 0011 | 22:g.48457077T>A | 279 | 0 | 37 |
| 2000 | X:g.99550325A>T | 114 | 291 | 0 |
| 0200 | 1:g.217814113A>T | 145 | 5 | 0 |
| 0020 | 8:g.102712231C>G | 254 | 0 | 0 |
| 0002 | 22:g.45100372C>G | 219 | 2 | 0 |
| 2200 | 10:g.10409000C>G | 70 | 0 | 0 |
| 2020 | 1:g.171704133C>G | 1 | 0 | 0 |
| 2002 | 22:g.43850796C>G | 72 | 1 | 0 |
| 0220 | 6:g.157990052C>G | 0 | 0 | 0 |
| 0202 | 22:g.37570268C>G | 0 | 0 | 480 |
| 0100 | 7:g.48349235C>G | 205 | 49 | 16 |
| 0022 | 15:g.93340834C>G | 109 | 0 | 0 |
| 1200 | 3:g.178141108C>G | 169 | 1 | 0 |
| 1020 | 3:g.185008261T>A | 148 | 2 | 0 |
| 1002 | 4:g.64156357A>C | 259 | 0 | 0 |
| 0120 | 20:g.31227002T>A | 0 | 109 | 0 |
| 0102 | 13:g.29868349C>A | 0 | 217 | 3 |
| 0012 | 2:g.227702681C>G | 1 | 0 | 238 |
| 2100 | 5:g.14507135C>T | 41 | 0 | 38 |
| 2010 | 16:g.9849871G>C | 34 | 0 | 0 |
| 2001 | 11:g.94429444A>C | 2 | 95 | 1 |
| 0010 | 17:g.5541887C>T | 624 | 1 | 5 |
| 0210 | 2:g.233772291G>T | 0 | 276 | 0 |
| 0201 | 20:g.35384132C>G | 0 | 0 | 177 |
| 0021 | 16:g.88586510A>C | 0 | 0 | 4 |
| 0001 | 5:g.153696723T>A | 312 | 5 | 50 |
| 1100 | X:g.98451133A>T | 28 | 133 | 16 |
| 1010 | 7:g.151045210C>G | 0 | 0 | 0 |
| 1001 | 17:g.11784435G>C | 6 | 14 | 134 |
| 0110 | 7:g.128815876C>G | 0 | 10 | 82 |
| 0101 | 12:g.889170C>G | 0 | 31 | 464 |
| Positive Partition Accuracy | | **3368/3561 (94.6%)** | **1121/1318 (85.1%)** | **1617/1776 (91.0%)** |

**Table S9**. **Comparison of USE-PCR with alternative targeted detection methods for a 32-target rare SNV detection assay.** All methods are modeled with 6,000 haploid genome equivalents of input DNA, including 10 mutant molecules per SNV. Performance is evaluated based on expected measurement density and sensitivity using representative implementations: Illumina NextSeq 1000 for amplicon-based NGS, and QIAcuity (26k partitions, 24-well format) for singleplex, 4-plex, and USE-PCR digital assays.

| Metric | Singleplex dPCR  (1 target x 32 reactions) | Multiplex dPCR  (4 targets x 8 reactions) | Targeted Amplicon Sequencing (32 targets x 1 reaction) | USE-PCR  (32 targets x 1 reaction) |
| --- | --- | --- | --- | --- |
| Separate reactions required | 32 | 8 | 1 | 1 |
| Haploid genome input per reaction | 188 | 750 | 6000 | 6000 |
| Variant molecules per SNV per reaction | 0.31 | 1.3 | 10 | 10 |
| Informative measurements per SNV | 0-1  (most get 0) | ~1-2 | 10 deduplicated UMI reads | 10  digital countable |
| Measurements per reaction^1^ | 26,000 | 104,000 | 4.2M^2^ | 208,000 |
| Measurements per SNV (raw) | 26,000 | 26,000 | 130,000 | 5,000 |
| Per-SNV informative measurements | 26,000 | 26,000 | ~3000-10,000 | 5,000 |
| Error suppression required | No | No | Yes  (UMIs, duplex) | No |
| Measurement efficiency (informative measurements per raw measurment) | 1.0  High | 1.0  High | 0.02-0.08  Low | 1.0  High |
| Detection sensitivity | Poor (due to dilution) | Moderate | High (after UMI correction) | High |
| Sample input efficiency | Very low | Moderate | High | High |
| Cost per sample | High | Moderate | Moderate | Low |
| Instrument CapEx | Low | Low | High | Low |
| Hands-on time | Low | Low | High | Low |
| Turnaround time | Hours | Hours | Days | Hours |

^1^QIAcuity 24-well plate and QIAcuity Probe PCR Kit; NextSeq 1000/2000 100 cycle kit, 100M total reads

^2^ Assumes 100M reads and 24 separate sequencing libraries on the run.

Table S10. Sequence information for the SNV detection primers for HCC1143, HCC1187, and HCC1395.

File: USE-PCR_supplementary_table_S10.xlsx

**Table S11.** Primer and probe sequences for the probe-based PCR assay Tick Borne Pathogen Detection.

| **Target ID** | **Forward primer** | **Allele-specific probes** | **Reverse Primer** |
| --- | --- | --- | --- |
| APG_rpoB | GAAGTTTGATCGCAAGGTAA | ACATGTTGAAGCTCCATCACTTG | CCAACAGACCTAGCATGTAT |
| BBM_ospA | CAACAGTAGACAAGCTTGAG | ATGGATCTGGAGTACTTGAAGG | TTTACTTTTGTCAGCTTTTACG |
| BMC_cox1 | ACATACGCTGTATCGTGTA | CATTGCCTAGGACTACTCCAGT | ACAGATAGTCATAGCCTTCAT |
| BMI_glpQ | TGCTTTAAACAAGAAATGGGT | ATCATAGCTCACAGGGGTGCTA | AGCTTCTAAGGTATGTTCTGG |
| ECH_120kDA | GATTCCGGTTGTTGTAGAGA | TGTTTGCACCTTCATTTAATCCAATCGT | TTGTTCGCAAGTTTCACAAA |
| EEW_p28 | AACTGACTTTTCAATCATGCTA | CCCCTTATATATGTGCAGTGTTTGG | AAACATTGACACTAAATCAGCA |
| EML_p28-14 | ACAACAGATCCAGCTTTTGAA | TCAGGGTTCTCAGGAAGTATTGGT | ATTGTTGGTATGCAGCCTC |
| RFB_glpQ | GACGAATTAAAGAGAATAAGGGAAG | TCAAGGAAAATTAATAATGCTTGTTGG | GTATCCAAGGTCCAATTCCA |
| RIC_17KDa | AGGTACAGGAACACTTCTTG | ACTTGGTTCTCAATTCGGTAAGGG | AGTAATGCACCTACACCTAC |

**Table S12.** USE-PCR encoding layout for 10-target TBP detection assay. The probes in **Table S9** were modified with color-coded tags according to the layout below.

| rs ID | FAM | HEX | TAMRA | ROX | Cy5 |
| --- | --- | --- | --- | --- | --- |
| APG_rpoB | 1 |  |  |  |  |
| BBM_ospA | 2 |  |  |  |  |
| BMC_cox1 |  | 1 |  |  |  |
| BMI_glpQ |  | 2 |  |  |  |
| ECH_120kDA |  |  |  |  | 1 |
| EEW_p28 |  |  |  |  | 2 |
| EML_p28-14 |  |  |  | 2 |  |
| RFB_glpQ |  |  |  | 1 | 2 |
| RIC_17KDa |  |  | 1 |  | 1 |
| RPP |  |  | 2 |  |  |
